# Evaluation of Clinical and Safety Outcomes of Neoadjuvant Immunotherapy Combined With Chemotherapy for Patients With Resectable Esophageal Cancer: A Systematic Review and Meta-analysis

**DOI:** 10.1001/jamanetworkopen.2022.39778

**Published:** 2022-11-02

**Authors:** Fan Ge, Zhenyu Huo, Xiuyu Cai, Qiyuan Hu, Wenhao Chen, Guo Lin, Ran Zhong, Zhending You, Rui Wang, Yi Lu, Runchen Wang, Qinhong Huang, Haotian Zhang, Aiqi Song, Caichen Li, Yaokai Wen, Yu Jiang, Hengrui Liang, Jianxing He, Wenhua Liang, Jun Liu

**Affiliations:** 1 Department of Thoracic Surgery and Oncology, China State Key Laboratory of Respiratory Disease and National Clinical Research Center for Respiratory Disease, the First Affiliated Hospital of Guangzhou Medical University, Guangzhou, China; 2 First Clinical School, Guangzhou Medical University, Guangzhou, China; 3 Nanshan School, Guangzhou Medical University, Guangzhou, China; 4 Department of General Internal Medicine, Sun Yat-sen University Cancer Centre, State Key Laboratory of Oncology in South China, Collaborative Innovation Centre for Cancer Medicine, Guangzhou, China; 5 The First Clinical Medical School, The First Hospital, Shanxi Medical University, Taiyuan, China; 6 School of Basic Medicine, Air Force Medical University, Xi’an, Shaanxi, China; 7 Department of Thoracic Oncology, Cancer Center and State Key Laboratory of Biotherapy, West China Hospital, Sichuan University, Chengdu, China; 8 West China School of Medicine, Sichuan University, Chengdu, China; 9 Department of Medical Oncology, Shanghai Pulmonary Hospital and Thoracic Cancer Institute, Tongji University School of Medicine, Shanghai, China

## Abstract

**Importance:**

A considerable number of clinical trials of neoadjuvant immunotherapy for patients with resectable esophageal cancer are emerging. However, systematic evaluations of these studies are lacking.

**Objective:**

To provide state-of-the-art evidence and normative theoretical support for neoadjuvant immunotherapy for locally advanced resectable esophageal cancer.

**Data Sources:**

PubMed, Embase, Cochrane Library, and ClinicalTrials.gov databases were searched for relevant original articles and conference proceedings that were published in English through April 1, 2022.

**Study Selection:**

Published phase 2 or 3 clinical trials that included patients with resectable stage I to IV esophageal cancer who received immune checkpoint inhibitors (ICIs) before surgery as monotherapy or in combination with other therapies.

**Data Extraction and Synthesis:**

The Preferred Reporting Items for Systematic Reviews and Meta-analyses and the Meta-analysis of Observational Studies in Epidemiology guidelines for meta-analysis were followed to extract data. A random-effects model was adopted if the heterogeneity was significant (*I*^2^ statistic >50%); otherwise, the common-effects model was used. Data analyses were conducted from April 2 to 8, 2022.

**Main Outcomes and Measures:**

Pathological complete response (pCR) rate and major pathological response (MPR) rate were considered to be the primary outcomes calculated for the clinical outcomes of neoadjuvant immunotherapy. Incidence of treatment-related severe adverse events was set as the major measure for the safety outcome. The rate of R0 surgical resection was summarized. Subgroup analyses were conducted according to histologic subtype and ICI types.

**Results:**

A total of 27 clinical trials with 815 patients were included. Pooled rates were 31.4% (95% CI, 27.6%-35.3%) for pCR and 48.9% (95% CI, 42.0-55.9%) for MCR in patients with esophageal cancer. In terms of safety, the pooled incidence of treatment-related severe adverse events was 26.9% (95% CI, 16.7%-38.3%). Most patients achieved R0 surgical resection (98.6%; 95% CI, 97.1%-99.6%). Regarding histologic subtypes, the pooled pCR rates were 32.4% (95% CI, 28.2%-36.8%) in esophageal squamous cell carcinoma and 25.2% (95% CI, 16.3%-35.1%) in esophageal adenocarcinoma. The pooled MPR rate was 49.4% (95% CI, 42.1%-56.7%) in esophageal squamous cell carcinoma.

**Conclusions and Relevance:**

This study found that neoadjuvant immunotherapy with chemotherapy had promising clinical and safety outcomes for patients with resectable esophageal cancer. Randomized clinical trials with long-term follow-up are warranted to validate the findings and benefits of ICIs.

## Key Points

**Question:**

What benefits are associated with neoadjuvant immunotherapy for patients with locally advanced resectable esophageal cancer?

**Findings:**

This systematic review and meta-analysis of 27 clinical trials with 815 patients found promising clinical and safety outcomes of neoadjuvant immunotherapy combined with chemotherapy in resectable esophageal cancer, providing clinical evidence to support the prospective wide application of this treatment option.

**Meaning:**

Findings of this study suggest the need for randomized clinical trials with long-term follow-up to validate the benefits of immune checkpoint inhibitors.

## Teaser

This systematic review and meta-analysis examines recent or ongoing nonrandomized clinical trials involving patients with esophageal cancer who received immune checkpoint inhibitors with or without other therapies.

## Introduction

Esophageal cancer is associated with the following distinct characteristics: multidisciplinary intervention requirements, substantially decreased quality of life, and poor outcome. According to estimates of GLOBOCAN 2020, esophageal cancer was the eighth most commonly diagnosed malignant neoplasm and the sixth most common cause of cancer death globally in 2020, accounting for 1 in every 20 cancer deaths.^1^

Surgery remains the mainstay for early-stage esophageal squamous cell carcinoma (ESCC) or esophageal adenocarcinoma (EAC).^2^ For locally advanced ESCC, the National Comprehensive Cancer Network and the Chinese Society of Clinical Oncology guidelines both recommended chemoradiotherapy (CRT) as the standard approach.^3,4^ Randomized clinical trials (RCTs) have demonstrated the extended overall survival (OS) of preoperative CRT compared with surgery alone.^5,6^ Resulting in a rather high pathological complete response (pCR) of nearly 40%, the NEOCRTEC_50_10 (Neoadjuvant Chemoradiotherapy for Esophageal Cancer 5010) and CROSS (Chemoradiotherapy for Esophageal Cancer Followed by Surgery Study) trials have set the neoadjuvant CRT (nCRT) as the standard treatment for locally advanced cases.^7,8^ The CROSS study demonstrated the absence of apparent adverse implication of nCRT for health-related quality of life compared with surgery only, which further supported the feasibility of nCRT in locally advanced ESCC.^9^ However, the expected long-term outcome for patients with ESCC remains poor. Radiotherapy-induced complications are associated with not only increased surgical difficulty but also reduced patient quality of life, which could ultimately be a factor in worse outcome for patients with ESCC.^10^ The 5-year OS rate among patients who received nCRTs and other surgeries was approximately 50%, and locoregional or distant metastasis incidence remained high.^11^

In 2020, KEYNOTE-590 (Randomized, Double-Blind, Placebo-Controlled Phase III Clinical Trial of Pembrolizumab in Combination With Cisplatin and 5-Fluorouracil Versus Placebo in Combination With Cisplatin and 5-Fluorouracil as First-Line Treatment in Subjects With Advanced/Metastatic Esophageal Carcinoma) was the first study of immunotherapy to report significantly improved OS in patients with localized or metastatic esophageal cancer worldwide.^12^ Results of this trial not only demonstrated improved OS, progression-free survival (PFS), duration of response, and objective response rate (ORR) associated with pembrolizumab combined with platinum and 5-fluorouracil, compared with first-line treatment with platinum-based chemotherapy alone, but also suggested that the safety data were comparable to those from standard chemotherapy.^13^ The KEYNOTE-590 study has rewritten the guidelines for the diagnosis and treatment of esophageal cancer and has changed the clinical practice in the treatment of advanced esophageal cancer worldwide.^11^ In addition, the results suggested the application potential of immunotherapy combined with chemotherapy in neoadjuvant therapy for locally advanced esophageal cancer. Following the footprint of the KEYNOTE-590 trial, numerous clinical trials of immunotherapy-based neoadjuvant treatment for esophageal cancer were registered.^14,15^ Most of the trials are still ongoing, and only part of their data have been released at academic conferences. However, no systematic review of the outcomes of current neoadjuvant immunotherapy trials for locally advanced ESCC or EAC has been performed.

Using available published data, we conducted this systematic review and meta-analysis to evaluate the clinical and safety outcomes of neoadjuvant immunotherapy for patients with locally advanced resectable esophageal cancer. We aimed to provide state-of-the-art evidence and normative theoretical support for this treatment option.

## Methods

We followed the Preferred Reporting Items for Systematic Reviews and Meta-analyses (PRISMA) reporting guideline^16^ and the Meta-analysis of Observational Studies in Epidemiology (MOOSE) reporting guideline.^17^ The study protocol was registered in PROSPERO (identifier CRD42022322991).

### Search Strategy and Study Selection

A comprehensive search of PubMed, Embase, Cochrane Library, and ClinicalTrials.gov databases was performed to identify prospective studies on neoadjuvant immunotherapy in esophageal cancer that were published in English and reported before April 1, 2022. Medical Subject Headings were applied to search for the following terms: *esophageal cancer* (including *esophageal squamous cell carcinoma* and *esophageal adenocarcinoma*), *neoadjuvant*, *preoperative*, *programmed cell death 1 (PD-1)*, *programmed cell death ligand 1 (PD-L1)*, and *immunotherapy* (including all immune checkpoint inhibitors [ICIs] currently known). We also searched conference abstracts from the European Society for Medical Oncology, the American Society of Clinical Oncology, and the American Association for Cancer Research up to April 1, 2022. eTable 1 in the Supplement presents the inclusion and exclusion criteria for available studies. When duplicate articles were identified, we included only the most updated version. Reference lists of the obtained literature were also searched for detailed information. All of the online searches were conducted using Microsoft Edge, version 104.0.1293.54 (Microsoft Corporation), and Google Chrome, version 104.0.5112.81 (Google LLC).

We selected published phase 2 or 3 clinical trials. These studies enrolled patients with resectable stage I to IV esophageal cancer who received ICIs as a monotherapy or combined with other therapies.

### Data Collection and Quality Assessment

With the predefined standardized form, 2 of us (F.G. and Z.H.) independently extracted the following items from each included study: first author, geographic location, year of publication or conference presentation, type of article, clinical trial phase, National Clinical Trial or Chinese Clinical Trial Registry identifier, main inclusion criteria, intervention model, masking method, types of neoadjuvant therapy, applied ICI drug, number of patients enrolled and undergoing surgery, patient information (including male to female ratio and mean or median age), pCR rate, major pathological response (MPR) rate, ORR, disease control rate (DCR), incidence of treatment-related severe adverse events (trSAE), R0 surgical resection (clinical and complete microscopic resection of the tumor) rate, and incidence of surgical complications. Race and ethnicity data were not collected because they were not available.

Each included study was reviewed several times to ensure that the data were neither incorrectly flagged nor missing. Any disagreement was resolved by consensus and arbitration by a panel of adjudicators (Y.W., Y.J., H.L., J.H., W.L., and J.L.). We also contacted the corresponding author of a study if relevant information was incomplete or not reported.

Because most clinical trials on neoadjuvant immunotherapy for patients with esophageal cancer were nonrandomized single-group series, there were no comparison groups. We used the Methodological Index for Nonrandomized Studies^18^ to assess the risk of bias in included studies. Two of us (F.G. and Z.H.) used criteria to independently score the quality of the studies and to judge whether the studies fulfilled the appropriate criteria for quantitative meta-analysis. Any discrepancies were resolved by consensus and arbitration by a panel of adjudicators (Y.W., Y.J., H.L., J.H., and W.L.).

### Outcome Measures

The pCR, defined as no evidence of residual tumor cells, is a widely used and powerful indicator of clinical outcome of neoadjuvant therapy. The MPR was defined as less than 10% of residual tumor cells. In the present study, the pCR and MPR rates were considered to be the primary outcomes, whereas the ORR and DCR were set as the secondary outcomes for assessing the clinical outcomes of neoadjuvant immunotherapy. According to the RECIST (Response Evaluation Criteria in Solid Tumors) guideline version 1.13,^19^ the outcome of solid tumors was divided into partial response, complete response, stable disease, and progressive disease. The ORR consisted of the proportion of complete response and partial response. The sum of complete response rate, partial response rate, and stable disease rate was defined as the DCR.

The trSAE was assessed by Common Terminology Criteria for Adverse Events, version 4.0. Incidences of trSAE and surgical complication were considered to be the primary measures for evaluating safety. The R0 surgical resection rate was set as the secondary measure for assessing the safety of neoadjuvant immunotherapy.

### Statistical Analysis

Given that the included studies were single-group clinical trials reporting proportions (eg, pCR and MPR), pooled estimates were obtained using binomial distribution for each outcome. The Freeman-Tukey double arcsine transformation was applied to stabilize variance, which aimed to normalize the outcomes before pooling. Proportion-based meta-analyses were applied using an inverse-variance weighting model (common-effects model) and the DerSimonian-Laird random-effects model based on inverse-variance weights to estimate the clinical and safety outcomes reported across clinical trials; estimates of the latter model would be adopted if there was significant heterogeneity (*I*^2^ statistic >50%). In the inverse-variance weighting method, the weight of each study is the inverse of the variance of the effect estimate, which aims to minimize the variance of the weighted mean.^20^ Data synthesis was performed in ESCC, and results analysis was performed in EAC. Subgroup analyses were conducted by ICI types applied in ESCC. Due to the limitation of the available data, it was difficult to conduct subgroup analyses of EAC. Therefore, only a preliminary analysis of EAC was performed with the available data. Heterogeneity across studies was identified using the Cochran Q Test and *I*^2^ test, and significant heterogeneity was considered to be *I*^2^ > 50%.

First, leave-1-out sensitivity analyses were performed to initially evaluate the potential confounding studies. Second, to explore the potential confounding factors, sensitivity analyses based on the stage of disease were conducted. Patients with relatively advanced disease were defined as having stage III to IVa. Third, Egger test was adopted to statistically analyze the publication biases.

All analyses were performed with the package meta, version 5.2-0, in RStudio, version 4.0.4 (RStudio). The meta and forestplot packages in R were used to generate plots. Tests were 2-tailed, and *P* < .05 was set as statistically significant. All analyses were conducted from April 2 to 8, 2022.

## Results

**Figure 1.**
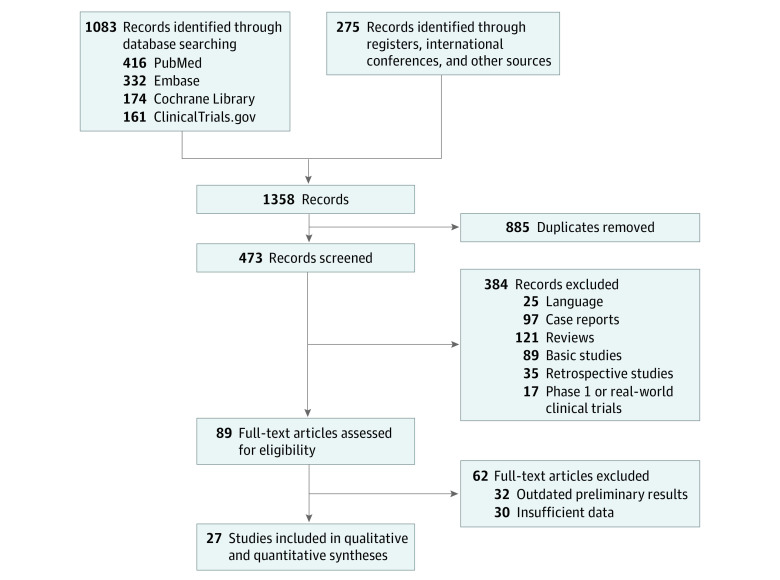
PRISMA Diagram for Study Identification and Selection

A total of 1358 records were retrieved through the initial search strategy. Of these articles, 885 duplicates were removed and 384 were excluded after screening the title and abstract. The remaining 89 full-text articles were evaluated for eligibility, 62 of which were removed because they provided outdated preliminary results or lacked valid data. Thus, 27 prospective studies^21,22,23,24,25,26,27,28,29,30,31,32,33,34,35,36,37,38,39,40,41,42,43,44,45,46,47^ were included in the final qualitative and quantitative synthesis (Figure 1). The geographic distribution of included studies is shown in eFigure 1 in the Supplement.

### Characteristics of Included Trials and Patients

Standardized characteristics of the included clinical trials are summarized in Table 1. Overall, the 27 trials included 815 patients with esophageal cancer being treated with neoadjuvant immunotherapy combined with chemotherapy who were available for analysis. All studies were open-label, phase 2 non-RCTs, and 25 of them were single-group^21,22,24,25,26,27,30,31,33,34,35,36,37,39,40,41,42,43,44,45,46,47^ and 2 were dual-group trials.^28,29^ Twenty-three trials^21,23,24,27,28,29,31,32,33,34,35,36,37,38,39,42,43,44,46,47^ enrolled patients with ESCC and applied anti–PD-1 antibody. Four trials^22,26,30,45^ enrolled patients with EAC, of which 2 used anti–PD-1 antibody^30,45^ and 2 applied anti–PD-L1 antibody.^22,26^

**Table 1.** Characteristics of Studies for Neoadjuvant Immunotherapy Combined With Chemotherapy in Resectable Esophageal Cancer

Study	Geographic location	Histologic subtype	Enrolled patients				No. of patients undergoing surgery	ICI	NCT or ChiCTR identifier	Model	Type of article
			Sample size, No.	Age, y	Male, No. (%)^a^	Clinical stage					
Lee et al,^21^ 2019	South Korea	ESCC	28	Mean (SD): 60 (NR)	NR	T1N1-2M0 or T2-4aN0-2M0	26	Pembrolizumab	NCT02844075	Single group	Conference abstract
van den Ende et al,^22^ 2019	Netherlands	EAC	40	Median (IQR): 63 (40-75)	35 (87.5%)	T1-4b, N0-N+, and M0	33	Atezolizumab	NCT03087864	Single group	Article
Gu et al,^23^ 2020	China	ESCC	17	Median (IQR): 65 (42-69)	13 (76.5%)	T1b-T3, N0-N+, and M0	15	Sintilimab	NCT03946969	Single group	Conference abstract
Zhang et al,^25^ 2020	China	ESCC	24	NR	NR	III or IVa	18	Toripalimab	ChiCTR1900027160	Single group	Conference abstract
Park et al,^24^ 2020	South Korea	ESCC	16	Median (IQR): 59 (57-66)	13 (81.3%)	T1N1-2M0 or T2-4aN0-2M0	16	Pembrolizumab	NCT02844075	Single group	Article
Zhang et al,^40^ 2021	China	ESCC	30	Mean (SD): 58 (7)	26 (86.7%)	III (90.0%) and IVa (10.0%)	23	Sintilimab	ChiCTR2100045659	Single group	Article
Duan et al,^27^ 2021	China	ESCC	23	Median (IQR): 64 (56-81)	21 (91.3%)	II (17.4%), III (73.9%), and IVa (8.7%)	17	Sintilimab	ChiCTR2100048917	Single group	Article
Xing et al,^28^ 2021	China	ESCC	15	Mean (SD): 64 (6)	13 (86.7%)	II (20.0%), III (46.7%), and IVa (33.3%)	11	Toripalimab	NCT03985670	Dual group	Article
Huang et al,^29^ 2021	China	ESCC	23	Mean (SD): 59 (7)	21 (91.3%)	II (13.0%), III (60.9%), and IVa (26.1%)	21	Pembrolizumab	NR	Dual group	Article
Wu et al,^37^ 2021	China	ESCC	38	Median (IQR): 61 (57-75)	36 (94.7%)	III-IVa	38	Multiple^b^	NR	Single group	Article
He et al,^42^ 2022	China	ESCC	20	Median (IQR): 62 (52-72)	5 (25.0%)	III (80.0%) and IVa (20.0%)	16	Toripalimab	NCT04177797	Single group	Article
Ma et al,^32^ 2021	China	ESCC	24	Median (IQR): 61 (50-73)	NR	IIa-IIIb	7	Camrelizumab	ChiCTR2000033761	Single group	Conference abstract
Yan et al,^38^ 2021	China	ESCC	45	NR	NR	II-IVa	36	Tislelizumab	ChiCTR2000037488	Single group	Conference abstract
Wang et al,^35^ 2021	China	ESCC	26	Mean (SD): 63 (NR)	17 (65.4%)	T2-4aN0-3M0	12	Camrelizumab	NCT03917966	Single group	Conference abstract
Zhang et al,^41^ 2021	China	ESCC	40	NR	NR	II-III	40	Sintilimab	ChiCTR1900026593	Single group	Conference abstract
Wang,^36^ 2021	China	ESCC	30	NR	NR	T1N2M0 or T2-3N0-2M0	24	Camrelizumab	ChiCTR1900023880	Single group	Conference abstract
Li et al,^30^ 2021	China	EAC	36	Mean (SD): 60 (NR)	24 (66.7%)	T2, N0-N+, and M0	28	Toripalimab	NCT04354662	Single group	Conference abstract
Liu et al,^31^ 2021	China	ESCC	23	NR	NR	T14a, N0-N+, and M0	18	Toripalimab	ChiCTR1900025318	Single group	Conference abstract
Yang et al,^47^ 2022	China	ESCC	23	Mean (SD): 59 (10)	22 (95.7%)	III (34.8%) and IVa (65.2%)	20	Camrelizumab	ChiCTR2000028900	Single group	Article
Shang et al,^33^ 2021	China	ESCC	42	NR	NR	T3N1M0 or T1-3N2M0	29	Pembrolizumab	NCT04389177	Single group	Conference abstract
Liu et al,^43^ 2022	China	ESCC	56	Median (IQR): 61 (40-70)	42 (75.0%)	II (23.2%), III (67.9%), and IVa (8.9%)	51	Camrelizumab	NCT04225364	Single group	Article
Shen et al,^34^ 2021	China	ESCC	28	Median (IQR): 62 (48-79)	27 (96.4%)	T1N1-3M0 or T2-4aN0-3M0	27	Multiple^c^	NR	Single group	Article
Yang et al,^39^ 2021	China	ESCC	12	Median (IQR): 56 (50-65)	7 (58.3%)	II (16.7%), III (66.7%), and IVa (16.7%)	12	Camrelizumab	ChiCTR2000029807	Single group	Article
Athauda et al,^26^ 2021	Germany	EAC	15	Median (IQR): 63 (25-73)	NR	T1-3N0-N2M0	15	Avelumab	NCT03399071	Single group	Conference abstract
Sun et al,^45^ 2022	US	EAC	35	Median (IQR): 65 (44-86)	28 (80.0%)	T1N1-3M0 or T2-3N0-3M0	26	Pembrolizumab	NCT03488667	Single group	Conference abstract
Liu et al,^44^ 2022	China	ESCC	60	Median (IQR): 65 (48-74)	50 (83.3%)	III (85.0%) and IVa (15.0%)	51	Camrelizumab	ChiCTR1900026240	Single group	Article
Xu et al,^46^ 2022	China	ESCC	46	NR	NR	II (23.9%), III (71.7%), and IVa (4.3%)	46	Camrelizumab	NCT04506138	Single group	Conference abstract

Abbreviations: ChiCTR, Chinese Clinical Trial Registry; EAC, esophageal adenocarcinoma; ESCC, esophageal squamous cell carcinoma; ICI, immune checkpoint inhibitor; NCT, National Clinical Trial; NR, not reported.

^a^ Only male sex data are presented due to the following reasons: (1) most patients with esophageal cancer were male, (2) reliability of data sources had to be ensured, and (3) most included studies presented only male sex data.

^b^ Camrelizumab, pembrolizumab, or sintilimab.

^c^ Camrelizumab, pembrolizumab, or nivolumab.

Table 1 shows the demographic characteristics of the included population, such as age, sex, and clinical stage. The methodologic quality of the included studies is presented in eFigure 2 in the Supplement. The specific scoring rules of these studies are provided in eTable 2 in the Supplement.

### Clinical and Safety Outcomes

**Figure 2.**
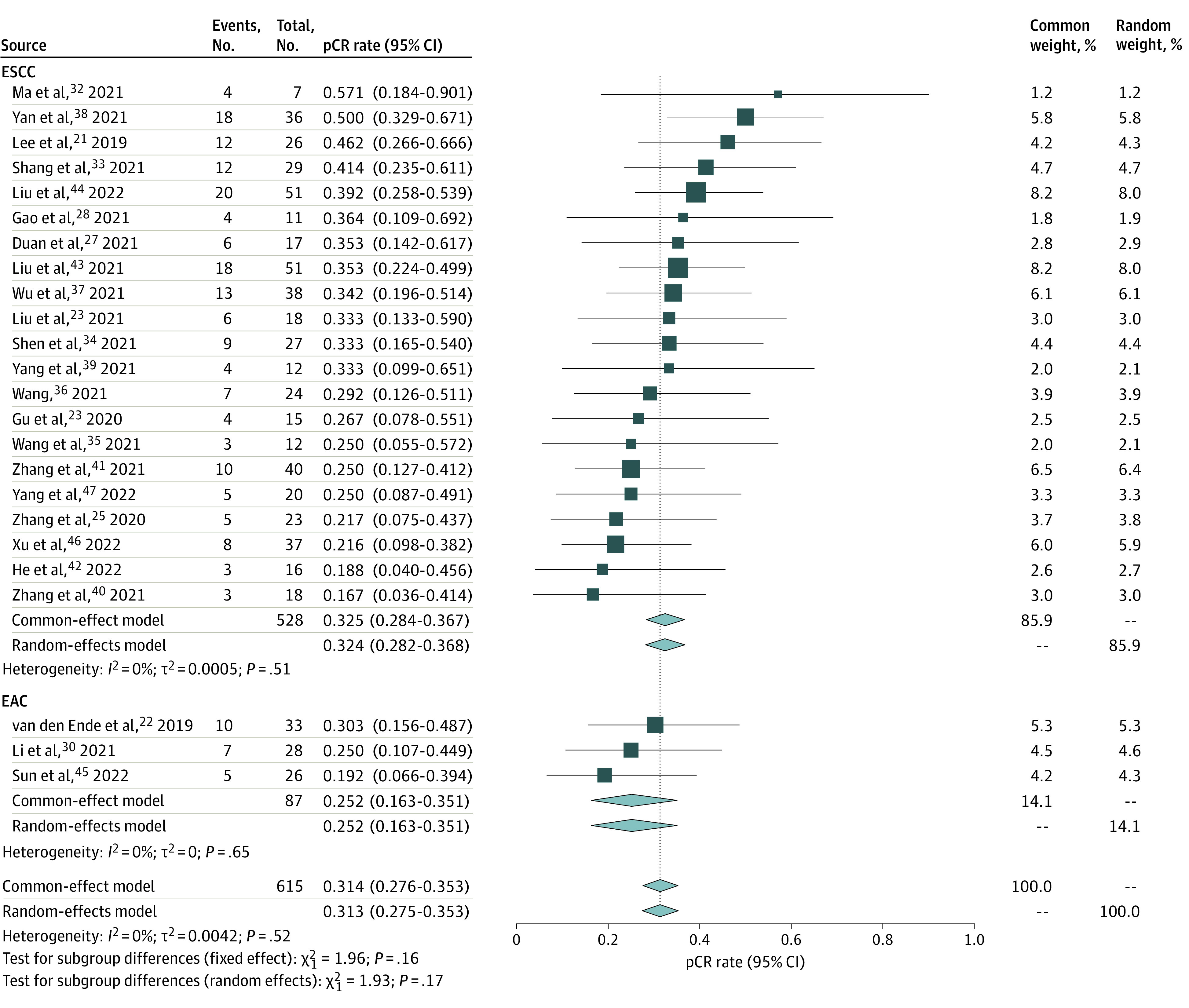
Forest Plot of Pathological Complete Response (pCR) of Neoadjuvant Immunotherapy Combined With Chemotherapy EAC indicates esophageal adenocarcinoma; ESCC, esophageal squamous cell carcinoma.

**Figure 3.**
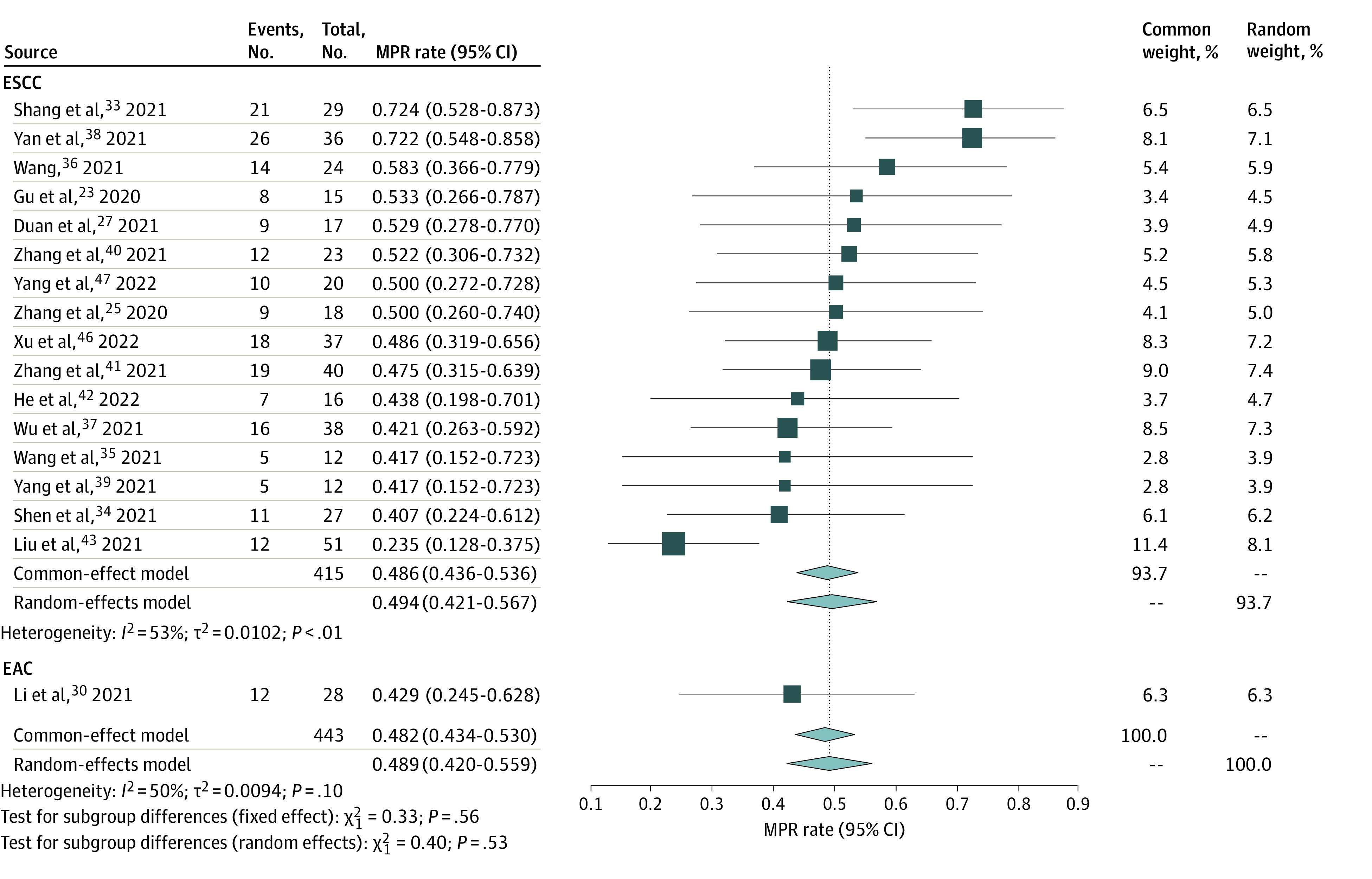
Forest Plot of Major Pathological Response (MPR) of Neoadjuvant Immunotherapy Combined With Chemotherapy EAC indicates esophageal adenocarcinoma; ESCC, esophageal squamous cell carcinoma.

In the present study, neoadjuvant immunotherapy demonstrated promising results for patients with esophageal cancer. According to 24 trials^21,22,23,24,25,27,28,30,32,33,34,35,36,37,38,39,40,41,42,43,44,45,46,47^ with 615 patients with esophageal cancer, the pooled pCR rate was 31.4% (95% CI, 27.6%-35.3%), with no significant heterogeneity (Figure 2). Meanwhile, based on 17 trials,^23,25,27,30,33,34,35,36,37,38,39,40,41,42,43,46,47^ the pooled MPR rate was 48.9% (95% CI, 42.0%-55.9%), with mild heterogeneity (*I*^2^ = 50.3%; 95% CI, 13.1%-71.5%; *P* = .10) (Figure 3). In terms of safety, 14 trials,^23,26,27,29,32,34,36,38,40,42,44,45,46,48^ including 452 patients, reported the incidence of trSAE; the pooled level was 26.9% (95% CI, 16.7%-38.3%), with significant heterogeneity (*I*^2^ = 84.9%; 95% CI, 76.2%-90.4%; *P* < .001) (eFigure 9 in the Supplement). eTable 3 in the Supplement summarizes the incidence of different surgical complications reported in the included studies. Most patients achieved R0 surgical resection. The pooled R0 surgical resection rate, based on 18 studies,^23,27,28,29,31,33,34,35,37,38,39,40,41,42,44,46,47,48^ was 98.6% (95% CI, 97.1%-99.6%), with no evidence of significant heterogeneity (eFigure 9 in the Supplement).

A pooled analysis of 21 studies^21,23,25,27,28,32,33,34,35,36,37,38,39,40,41,42,43,44,46,47^ suggested that the pCR rate was 32.4% (95% CI, 28.2%-36.8%) in ESCC, with no evidence of significant heterogeneity (Figure 2). Furthermore, the pooled MPR rate, based on 16 studies,^23,25,27,33,34,35,36,37,38,39,40,41,42,43,46,47^ was 49.4% (95% CI, 42.1%-56.7%) in ESCC, with evidence of heterogeneity (*I*^2^ *=* 53.0%; 95% CI, 0.0%-68.1%; *P* = .007) (Figure 3). Using the RECIST guideline version 1.13, we analyzed the results of the imaging assessment after neoadjuvant therapy: the pooled ORR was 70.5% (95% CI, 62.0%-78.5%), with a complete response rate of 21.9% and partial response rate of 54.9% (eFigure 4 in the Supplement); a slight heterogeneity was observed (*I*^2^ *=* 51.2%; 95% CI, 5.7%-74.8%; *P* = .02). The pooled DCR was 99.2% (95% CI, 97.0%-100.0%), with no significant heterogeneity observed (eFigure 4 in the Supplement). In terms of the safety of neoadjuvant immunotherapy combined with chemotherapy in ESCC, the pooled incidence of trSAE was 22.7% (95% CI, 13.3%-33.5%), with evidence of significant heterogeneity (*I*^2^ *=* 83.6%; 95% CI, 72.7%-90.1%; *P* < .001) (eFigure 9 in the Supplement). Moreover, the R0 surgical resection rate achieved 98.0% (95% CI, 96.1%-99.4%), with no significant heterogeneity observed (eFigure 9 in the Supplement).

A pooled analysis of 3 studies^22,30,45^ suggested that the pCR rate was 25.2% (95% CI, 16.3%-35.1%) in EAC (Figure 2). No significant heterogeneity was found; thus, the common-effects model was adopted for analysis. However, only 1 study^30^ reported an MPR rate of 42.9% (95% CI, 24.5%-62.8%) in EAC (Figure 3). Based on 2 trials,^23,27,29,32,34,36,38,40,42,44,46,48^ the pooled incidence of trSAE was 56.0% (95% CI, 41.7%-69.9%) (eFigure 9 in the Supplement). Meanwhile, the R0 surgical resection rate was 100.0% (95% CI, 98.0%-100.0%) (eFigure 9 in the Supplement).

### Exploratory Subgroup Analysis

Exploratory subgroup analysis was performed to explore the potential association between ICI types and the multiple outcomes of neoadjuvant immunotherapy. Among the 23 included ESCC studies that applied anti–PD-1 antibody, 8 used camrelizumab,^32,35,36,39,43,44,46,47^ 4 used pembrolizumab,^21,27,29,33^ 4 used sintilimab,^23,27,40,41^ 4 used toripalimab,^42^ 1 used tislelizumab,^38^ and 2 used multiple drugs.^34,37^ Due to the limitations of available data, exploratory subgroup analyses were conducted only in ESCC. A comparison of outcomes in different ICIs is provided in Table 2 and eFigures 3 to 5 in the Supplement.

**Table 2.** Subgroup Analysis of Immune Checkpoint Inhibitor Types in Esophageal Squamous Cell Carcinoma

	Pembrolizumab				Sintilimab				Toripalimab				Tislelizumab				Camrelizumab			
	No.	Rate (95% CI), %	*I*^2^, %	*P* value	No.	Rate (95% CI), %	*I*^2^, %	*P* value	No.	Rate (95% CI), %	*I*^2^, %	*P* value	No.	Rate (95% CI), %	*I*^2^, %	*P* value	No.	Rate (95% CI), %	*I*^2^, %	*P* value
pCR	2	43.6 (30.5-57.2)	0	.73	4	26.1 (17.3-35.7)	0	.82	4	24.9 (14.4-36.9)	0	.53	1	50.0 (32.9-67.1)	NA	NA	8	31.7 (25.3-38.4)	0	.58
MPR	1	72.4 (52.8-87.3)	NA	NA	4	50.5 (40.2-60.9)	0	.97	2	47.1 (30.1-64.4)	0	.73	1	72.2 (54.8-85.8)	NA	NA	6	42.7 (30.6-53.3)	56	.05
CR	1	34.8 (16.4-57.3)	NA	NA	1	37.5 (8.5-75.7)	NA	NA	1	33. (13.3-59.0)	NA	NA	NA	NA	NA	NA	3	8.8 (3.6-15.9)	0	.42
PR	1	52.2 (30.6-73.2)	NA	NA	2	49.2 (12.1-86.8)	76	<.05	2	54.5 (8.3-96.4)	91	<.05	NA	NA	NA	NA	4	55.9 (37.1-73.9)	68	<.05
SD	1	8.7 (1.1-28.0)	NA	NA	2	28.4 (14.3-44.7)	0	.86	2	28.1 (15.1-43.1)	35	.21	NA	NA	NA	NA	4	25.5 (2.7-58.5)	90	<.05
ORR	1	87.0 (66.4-97.2)	NA	NA	2	66.2 (49.6-81.2)	0	.79	3	69.9 (58.2-80.5)	0	.42	NA	NA	NA	NA	4	62.8 (43.5-80.2)	69	<.05
DCR	1	95.7 (78.1-99.9)	NA	NA	2	96.2 (85.5-100)	3	.31	2	100 (95.5-100)	0	.92	NA	NA	NA	NA	4	98.4 (89.0-100)	69	<.05
trSAE	1	39.1 (19.7-61.5)	NA	NA	3	19.9 (2.5-45.9)	82	<.05	NA	NA	NA	NA	1	34.9 (21.0-50.9)	NA	NA	5	23.0 (7.3-43.6)	91	<.05
R0 surgical resection	2	100 (96.2-100)	0	.91	4	98.8 (94.5-100)	0	.66	3	97.8 (89.7-100)	33	.22	1	97.2 (85.5-99.9)	NA	NA	6	98.2 (91.5-100)	72	<.05

Abbreviations: CR, complete response; DCR, disease control rate; MPR, major pathological response; NA, not applicable; ORR, objective response rate; pCR, pathological complete response; PR, partial response; SD, stable disease; trSAE, treatment-related severe adverse events.

### Sensitivity Analysis and Publication Bias

To ensure that the combined outcomes were not severely altered by the specific trials, we conducted leave-1-out sensitivity analyses by successively omitting each study. The results suggested that the overall estimates remained consistent across these analyses (eFigure 6 in the Supplement). For example, omitting any study was not associated with fluctuations in the overall pCR rate. Meanwhile, the sensitivity analyses based on the stage of disease showed that heterogeneity decreased significantly in the relatively advanced subgroup, suggesting that disease stage is a potential source of heterogeneity (eFigure 7 in the Supplement). For example, no significant heterogeneity was observed in the pCR rate of the relatively advanced subgroup.

Egger tests were adopted, and Egger regression asymmetry plots were constructed to evaluate the possible publication bias in pooled analyses of the clinical and safety outcomes of neoadjuvant immunotherapy. As shown in eFigure 8 in the Supplement, publication biases were absent from the Egger regression asymmetry plots.

## Discussion

Since 2017, immunotherapy has been applied as a third-line to a second-line to a first-line treatment for patients with advanced esophageal cancer, showing the potential advantage of immunotherapy in the treatment of this disease. However, the clinical and safety outcomes of neoadjuvant immunotherapy in esophageal cancer have not been comprehensively evaluated. To our knowledge, this systematic review and meta-analysis of clinical trials on neoadjuvant immunotherapy for esophageal cancer was the first of its kind. We believe the findings, based on 27 clinical trials involving 815 patients, provide evidence and guidance for the application of neoadjuvant immunotherapy in esophageal cancer.

In this meta-analysis, the overall pooled rate of pCR for neoadjuvant immunotherapy was 31.4%, which demonstrated the promising results of neoadjuvant immunotherapy for patients with esophageal cancer. Especially in ESCC, the synthesized pCR rate reached 32.5% and the highest pCR rate was 57.1% in the study by Ma et al^32^ reported in an American Society of Clinical Oncology conference abstract. As for neoadjuvant chemotherapy (NCT), some studies reported a 1.9% to 9.0% rate of pCR.^49,50^ The pCR rate for nCRT reported by the CROSS study^51^ was 24.0%. These outcomes indicated that the pCR for neoadjuvant immunotherapy was superior to the pCR for NCT and nCRT.

Regarding the MPR, latest data from clinical trials presented a 33.3% MPR rate for NCT,^52^ whereas the pooled MPR rate for neoadjuvant immunotherapy reached 48.9% in 17 trials,^23,25,27,30,33,34,35,36,37,38,39,40,41,42,43,46,47^ and the highest MPR rate was 72.4% in a study by Shang et al.^33^ Such outcomes could provide sufficient evidence for the feasibility of neoadjuvant immunotherapy. However, because most of the included clinical trials have not revealed long-term follow-up results and complete survival data, we could not ascertain the benefit of neoadjuvant immunotherapy for extended survival. Only the study by Duan et al^27^ showed that the mean disease-free survival was 13.8 months as of the last follow-up date among 17 patients. Future studies of PFS and OS data may identify the long-term survival benefit of neoadjuvant immunotherapy.

On the other hand, the safety analysis demonstrated the advantages of neoadjuvant immunotherapy. Several clinical trials suggested that immunotherapy or immunotherapy combined with chemotherapy was not significantly associated with increased incidence of trSAE.^13,53,54^ The pooled incidence of trSAE was 26.9%, with no treatment-related deaths observed, demonstrating good tolerability. In addition, because most ICIs had been investigated in completed clinical trials and adopted to treat various advanced solid tumors, there were ample clinical experience and consensus regarding the identification and effective handling of adverse events. Given that most studies only reported the incidence of specific, instead of overall, surgical complications, we did not summarize the overall rates to avoid false reduction in total surgical complication rates due to inadequate data. The incidence of specific surgical complications was also lower for immunotherapy combined with chemotherapy compared with NCT.^48,55,56^ The fatal surgical complications were rare: only 2 deaths (0.3%) from acute respiratory distress syndrome were reported.^24^ The R0 surgical resection rate was 98.6%, which was much higher than the 81.7% to 86.6% for NCT,^48,56^ suggesting that neoadjuvant ICIs were not associated with reduced probability of surgical resection or accelerated tumor progression to the unresectable point. These promising results suggested the acceptable safety of neoadjuvant immunotherapy.

There are still no biomarkers that can accurately estimate the clinical outcomes of immunotherapy for patients with esophageal cancer. According to the results of KEYNOTE-181 (Study of Pembrolizumab Versus Investigator's Choice Standard Therapy for Participants With Advanced Esophageal/Esophagogastric Junction Carcinoma That Progressed After First-Line Therapy) clinical trial,^57^ pembrolizumab was associated with prolonged OS, compared with chemotherapy, in patients with combined positive score of PD-L1 of 10 or higher, with fewer treatment-related adverse events. However, both ESCORT (Evaluation of Efficacy, Quality of Life and Cost Effectiveness of Short-course Radiotherapy Followed by Capecitabine Plus Oxaliplatin chemotheRapy and TME for High-risk Rectal Cancer)^58^ and ATTRACTION-3 (Nivolumab Versus Chemotherapy in Patients With Advanced Oesophageal Squamous Cell Carcinoma Refractory or Intolerant to Previous Chemotherapy)^59^ trials showed that patients benefited from immunotherapy regardless of PD-L1 expression levels. Similarly, the association between PD-L1 expression levels and pathological responses in neoadjuvant immunotherapy has remained controversial. Several studies indicated that neither the tissue polypeptide specific antigen nor combined positive score of PD-L1 expression was associated with pathologic response.^42,43,60,61^ However, based on data from the study by Yang et al,^62^ compared with the non-pCR group, the pCR group had significantly higher levels of PD-L1 and tumor mutation burden before treatment. This finding supported that the antitumor activity was associated with the increase of PD-L1 levels and tumor mutation burden in the primary tumor. Overall, the expression of PD-L1 and tumor mutation burden level abundance have attracted great attention in immunotherapy.^63,64,65,66^ However, due to the deficiency of research data, we cannot perform a meta-regression analysis to factor in these potential biomarkers. Follow-up clinical trials are warranted to ascertain whether these 2 biomarkers can be applied to esophageal cancer. It is important to investigate other effective biomarkers.

The differences in outcomes between various neoadjuvant treatment modalities are also worth exploring. According to a meta-analysis of studies involving 4529 patients with esophageal cancer, compared with NCT, nCRT provided a higher pCR rate, higher R0 surgical resection rate, and lower local recurrence and distant metastasis rates but no increase in 5-year survival.^67^ Based on trials involving 4563 patients with esophageal cancer, a network meta-analysis observed no differences between NCT and nCRT regarding OS and disease-free survival.^68^ Due to the limitations of available data, we could not explore the differences in results between neoadjuvant immunotherapy combined with chemotherapy and neoadjuvant immunotherapy combined with CRT. Previous studies have shown that the proportion of PD-L1–expressing immune cells and high CD8+ tumor cell densities significantly increased in patients with ESCC after NCT.^69^ In addition, cell experiments found that the expression of PD-L1 on the ESCC cell surface was significantly increased in a dose-dependent manner after 24 hours of radiation exposure.^70^ These findings suggest that chemotherapy and radiotherapy may achieve sensitization to immunotherapy by inducing increased expression of PD-L1 on cells. Therefore, in the context of immunotherapy, it is worth exploring the differences in clinical and safety outcomes between different neoadjuvant treatment modalities.

Several challenges need to be addressed before neoadjuvant immunotherapy for patients with esophageal cancer can become the standard treatment. First, the optimal strategy for neoadjuvant immunotherapy in esophageal cancer has not been established; large RCTs are warranted to compare whether immunotherapy should be applied alone or in combination with chemotherapy or radiotherapy. Second, current evidence for neoadjuvant immunotherapy in EAC remains relatively sparse; more clinical trials of neoadjuvant immunotherapy in EAC are still needed, especially in Europe and the US where the incidence of EAC is relatively high. Third, given that neoadjuvant therapy combined with adjuvant therapy has been shown to have satisfactory clinical outcomes and longer survival in other tumors, the feasibility of this combination is worth exploring in esophageal cancer. Moreover, a comparison of the differences in the clinical outcomes of different ICIs should be conducted. It is also important to investigate the biomarkers that can accurately estimate the clinical outcomes of immunotherapy in esophageal cancer. Fourth, the association between pathological response and survival in esophageal cancer deserves further investigation.

### Limitations

This study has several limitations. First, given that some included studies have not achieved their primary end points, the complete protocols and data of those studies were not available, making it difficult to investigate some survival indicators (such as PFS and OS). However, we are confident that the results presented herein would suffice until long-term results and follow-up data become available from future RCTs, which can link the pathological response in neoadjuvant immunotherapy to OS and PFS and explore the clinical and safety outcomes of neoadjuvant immunotherapy. Second, only 4 included studies reported the outcomes of EAC. Due to the limitations of available data, we could not conduct a comprehensive analysis of EAC. Because of the diversity between ESCC and EAC in pathological features and molecular characteristics, which may lead to different responses to immunotherapy, further subgroup analyses are needed as more data on EAC become available in the future. In the present study, the lack of adequate studies on EAC also placed a higher weight on the ESCC group, which may bias the pooled estimates toward ESCC group when generating the overall results. Hence, the overall results need to be interpreted with caution because they are not fully applicable to EAC. To generate more applicable results, we performed subgroup analyses to separate EAC from ESCC groups, which may help us better understand the differences in the response to neoadjuvant immunotherapy according to histologic subtypes.

Third, due to the limitations of available data, we could not perform a sensitivity analysis of the stage of disease in the measure of trSAE incidence. However, the disease stage may be a potential source of heterogeneity in the MPR and R0 surgical resection rates. In terms of combination therapy, there are few studies of neoadjuvant immunotherapy combined with CRT, which adds difficulty in exploring the difference in results between neoadjuvant immunotherapy plus chemotherapy and neoadjuvant immunotherapy plus CRT. Fourth, although exploratory subgroup analyses according to ICI drug types were performed, the number of included studies was still relatively small, leading to smaller sample sizes for certain drugs. This factor may also be one of the reasons for the significant heterogeneity in the subgroup analyses. In addition, most included studies were from China, which may limit the generalizability of the results of the present study. It is reassuring that numerous clinical trials of neoadjuvant immunotherapy for esophageal cancer have been approved or are enrolling patients from various locations (eg, Europe and US). We look forward to having more diverse data in the future, which would expand the generalizability of the results.

Fifth, all included studies were open label and nonrandomized, which may have led to the instability and bias of the findings. However, sensitivity analysis and Egger test showed robust results, and no publication bias was observed. Neoadjuvant immunotherapy for esophageal cancer is still under exploration, with the most recent published studies being single-group non-RCTs. Randomized clinical trials have a strict design, which requires a lot of resources and a long study period. Therefore, the large-scale RCTs are difficult to carry out with inadequate pretrial evidence to support the feasibility of a novel therapeutic intervention. To our knowledge, this study was the first comprehensive analysis to include current clinical trials, and thus we aimed to provide such evidence. Meanwhile, we also look forward to future RCTs with larger sample sizes and complete data to validate the results of this study.

## Conclusions

This systematic review and meta-analysis of 27 nonrandomized clinical trials demonstrated the promising clinical and safety outcomes of neoadjuvant immunotherapy combined with chemotherapy for patients with resectable esophageal cancer, providing clinical evidence to support the prospective wide application of this treatment option. The findings of this study serve as a basis for future research, and RCTs with long-term follow-up are warranted to validate the findings and the benefits of ICIs.

## Notes

Supplement. **eTable 1.** Inclusion and Exclusion Criteria **eTable 2.** Methodological Quality Assessment of Included Studies: MINORS **eTable 3.** Summary of Surgical Complications in Included Studies **eFigure 1.** The Geographical Distribution of Included Studies **eFigure 2.** Methodological Quality Assessment of Included Studies **eFigure 3.** Forest Plot of the Primary Outcomes for Clinical Outcomes in Neoadjuvant Immunotherapy Combined With Chemotherapy in ESCC Stratified by ICI Types **eFigure 4.** Forest Plot of the Secondary Outcomes for Clinical Outcomes in Neoadjuvant Immunotherapy Combined With Chemotherapy in ESCC Stratified by ICI Types **eFigure 5.** Forest Plot of the Outcomes for Safety in Neoadjuvant Immunotherapy Combined With Chemotherapy in ESCC Stratified by ICI Types **eFigure 6.** Sensitivity Analyses of the Outcomes by Repeating the Pooled Analyses With One Study Omitted at a Time **eFigure 7.** Sensitivity Analyses Based on the Stage of Disease in ESCC **eFigure 8.** Egger’s Tests of the Outcomes to Detect Publication Bias **eFigure 9.** Forest Plot of Safety Outcomes of Neoadjuvant Immunotherapy Combined With Chemotherapy
