# Supplementary material for: Evaluation of Clinical and Safety Outcomes of Neoadjuvant Immunotherapy Combined With Chemotherapy for Patients With Resectable Esophageal Cancer: A Systematic Review and Meta-analysis
Source: JAMA Netw Open. 2022 Nov 2;5(11):e2239778. doi: 10.1001/jamanetworkopen.2022.39778 (PMC9631099; doi:10.1001/jamanetworkopen.2022.39778)
Supplement: Supplement. — eTable 1. Inclusion and Exclusion Criteria eTable 2. Methodological Quality Assessment of Included Studies: MINORS eTable 3. Summary of Surgical Complications in Included Studies eFigure 1. The Geographical Distribution of Included Studies eFigure 2. Methodological Quality Assessment of Included Studies eFigure 3. Forest Plot of the Primary Outcomes for Clinical Outcomes in Neoadjuvant Immunotherapy Combined With Chemotherapy in ESCC Stratified by ICI Types eFigure 4. Forest Plot of the Secondary Outcomes for Clinical Outcomes in Neoadjuvant Immunotherapy Combined With Chemotherapy in ESCC Stratified by ICI Types eFigure 5. Forest Plot of the Outcomes for Safety in Neoadjuvant Immunotherapy Combined With Chemotherapy in ESCC Stratified by ICI Types eFigure 6. Sensitivity Analyses of the Outcomes by Repeating the Pooled Analyses With One Study Omitted at a Time eFigure 7. Sensitivity Analyses Based on the Stage of Disease in ESCC eFigure 8. Egger’s Tests of the Outcomes to Detect Publication Bias eFigure 9. Forest Plot of Safety Outcomes of Neoadjuvant Immunotherapy Combined With Chemotherapy [file jamanetwopen-e2239778-s001.pdf]

Supplemental Online Content

Ge F, Huo Z, Cai X, et al. Evaluation of clinical and safety outcomes of neoadjuvant immunotherapy combined with chemotherapy for patients with resectable esophageal cancer: a systematic review and meta-analysis. *JAMA Netw Open*. 2022;5(11):e2239778. doi:10.1001/jamanetworkopen.2022.39778

eTable 1. Inclusion and Exclusion Criteria

eTable 2. Methodological Quality Assessment of Included Studies: MINORS

eTable 3. Summary of Surgical Complications in Included Studies

eFigure 1. The Geographical Distribution of Included Studies

eFigure 2. Methodological Quality Assessment of Included Studies

eFigure 3. Forest Plot of the Primary Outcomes for Clinical Outcomes in Neoadjuvant Immunotherapy Combined With Chemotherapy in ESCC Stratified by ICI Types

eFigure 4. Forest Plot of the Secondary Outcomes for Clinical Outcomes in Neoadjuvant Immunotherapy Combined With Chemotherapy in ESCC Stratified by ICI Types

eFigure 5. Forest Plot of the Outcomes for Safety in Neoadjuvant Immunotherapy Combined With Chemotherapy in ESCC Stratified by ICI Types

eFigure 6. Sensitivity Analyses of the Outcomes by Repeating the Pooled Analyses With One Study Omitted at a Time

eFigure 7. Sensitivity Analyses Based on the Stage of Disease in ESCC

eFigure 8. Egger's Tests of the Outcomes to Detect Publication Bias

eFigure 9. Forest Plot of Safety Outcomes of Neoadjuvant Immunotherapy Combined With Chemotherapy

This supplemental material has been provided by the authors to give readers additional information about their work.

© 2022 Ge F et al. *JAMA Network Open.*

**eTable 1**. Inclusion and Exclusion Criteria.	
Inclusion Criteria	Exclusion Criteria
1. Published as full-length articles in English	1. Studies reporting inoperable, or metastatic disease
2. Phase II/III clinical trials reporting resectable stage I-IV esophageal cancer (including ESCC and EAC) confirmed by tissue	2. Included patients had received prior immunotherapy or chemoradiotherapy
3. Studies that included patients who received ICIs preoperatively as monotherapy or in combination with other therapies (including chemotherapy, and chemoradiotherapy)	3. Phase I clinical trials or real-world studies
4. Studies reporting the complete protocol, recruited patient data and at least one primary or secondary clinical outcome, such as pCR rate, MPR rate, ORR, DCR, the incidence of side effects were more than grade 3 tr-SAE, R0 surgical resection rate, and the incidence of surgical complications	4. Studies reporting invalid data for the efficacy and safety of neoadjuvant immunotherapy
	5. Studies that violated any of the inclusion criteria above. The reviewers further exclude case reports, systematic reviews or meta-analyses, and cell culture or animal studies, but not conference summaries
ESCC, esophageal squamous cell carcinoma; EAC, esophageal adenocarcinoma; ICI, immune checkpoint inhibitor; pCR, pathological complete response; MPR, major pathological response; ORR, objective response rate; DCR, disease control rate; tr-SAE, treatment-related severe adverse events;	

© 2022 Ge F et al. *JAMA Network Open.*

**eTable 2.** Methodological quality assessment of included studies: MINORS											
Study	1	2	3	4	5	6	7	8	9*	11*	12*	Score#
Lee et al,[21] 2019	2	2	1	2	2	2	2	2	NA	NA	NA	15
van den Ende et al,[22] 2019	2	2	2	1	2	2	2	1	NA	NA	NA	14
Gu et al,[23] 2020	2	2	2	2	2	2	2	1	NA	NA	NA	15
Zhang et al,[25] 2020	2	2	1	2	2	2	2	2	NA	NA	NA	15
Park et al,[24] 2020	2	2	1	1	2	1	2	1	NA	NA	NA	12
Zhang et al,[40] 2021	2	2	1	2	2	2	2	2	NA	NA	NA	15
Duan et al,[27] 2021	2	2	1	2	2	2	2	2	NA	NA	NA	15
Xing et al,[28] 2021	2	2	1	2	2	2	2	1	2	2	2	23
Huang et al,[29] 2021	2	2	1	1	2	2	2	1	2	2	2	22
Wu et al,[37] 2021	2	2	2	1	2	2	2	1	NA	NA	NA	14
He et al,[42] 2021	2	2	1	2	2	2	2	2	NA	NA	NA	15
Ma et al,[32] 2021	2	2	2	2	2	2	2	1	NA	NA	NA	15
Yan et al,[38] 2021	2	2	2	2	2	2	2	1	NA	NA	NA	15
Wang et al,[35] 2021	2	2	2	2	2	2	2	2	NA	NA	NA	16
Zhang et al,[41] 2021	2	2	2	2	2	2	2	1	NA	NA	NA	15

© 2022 Ge F et al. *JAMA Network Open.*

Wang et al,[36] 2021	2	2	2	2	2	2	2	1	NA	NA	NA	15
Li et al,[30] 2021	2	2	1	2	2	2	2	2	NA	NA	NA	15
Liu et al,[31] 2021	2	2	1	2	2	2	2	1	NA	NA	NA	14
Yang et al,[47] 2021	2	2	1	2	2	2	2	2	NA	NA	NA	15
Shang et al,[33] 2021	2	2	1	2	2	2	2	1	NA	NA	NA	14
Liu et al,[43] 2021	2	2	2	2	2	2	2	1	NA	NA	NA	15
Shen et al,[34] 2021	2	2	1	2	2	2	2	2	NA	NA	NA	15
Yang et al,[39] 2021	2	2	1	2	2	2	2	1	NA	NA	NA	14
Athauda et al,[26] 2021	2	2	1	1	2	2	2	1	NA	NA	NA	13
Sun et al,[45] 2022	2	2	1	2	2	2	2	1	NA	NA	NA	14
Liu et al,[44] 2022	2	2	1	2	2	2	2	2	NA	NA	NA	15
Xu et al,[46] 2022	2	2	2	2	2	2	2	1	NA	NA	NA	15

*, For comparative studies only.

#, Scores of at least 75% were considered high quality with low risk for bias; scores between 50% and 75% were considered medium risk for bias; scores of less than or equal to 50% were considered high risk for bias. For noncomparative studies, the maximum score was 16, while the maximum score for comparative studies was 24.

MINORS, Methodological index for non-randomized studies

© 2022 Ge F et al. *JAMA Network Open.*

Attachment: MINORS Evaluation Criteria	
**Methodological items for non-randomized studies**	Score#
1. **A clearly stated aim**: the question addressed should be precise and relevant in the light of available literature	
2. **Inclusion of consecutive patients**: all patients potentially fit for inclusion (satisfying the criteria for inclusion) have been included in the study during the study period (no exclusion or details about the reasons for exclusion)	
3. **Prospective collection of data**: data were collected according to a protocol established before the beginning of the study	
4. **Endpoints appropriate to the aim of the study**: unambiguous explanation of the criteria used to evaluate the main outcome which should be in accordance with the question addressed by the study. Also, the endpoints should be assessed on an intention-to-treat basis.	
5. **Unbiased assessment of the study endpoint**: blind evaluation of objective endpoints and double-blind evaluation of subjective endpoints. Otherwise the reasons for not blinding should be stated	
6. **Follow-up period appropriate to the aim of the study**: the follow-up should be sufficiently long to allow the assessment of the main endpoint and possible adverse events	
7. **Loss to follow up less than 5%**: all patients should be included in the follow up. Otherwise, the proportion lost to follow up should not exceed the proportion experiencing the major endpoint	
8. **Prospective calculation of the study size**: information of the size of detectable difference of interest with a calculation of 95% confidence interval, according to the expected incidence of the outcome event, and information about the level for statistical significance and estimates of power when comparing the outcomes	
Additional criteria in the case of comparative study	
9. **An adequate control group**: having a gold standard diagnostic test or therapeutic intervention recognized as the optimal intervention according to the available published data	
10. **Contemporary groups**: control and studied group should be managed during the same time period (no historical comparison)	
11. **Baseline equivalence of groups**: the groups should be similar regarding the criteria other than the studied endpoints. Absence of confounding factors that could bias the interpretation of the results	
12. **Adequate statistical analyses**: whether the statistics were in accordance with the type of study with calculation of confidence intervals or relative risk	
# The items are scored 0 (not reported), 1 (reported but inadequate) or 2 (reported and adequate). The global ideal score being 16 for non-comparative studies and 24 for comparative studies.	

© 2022 Ge F et al. *JAMA Network Open.*

eTable 3. Summary of surgical complications in included studies.

	N	Overall surgical complication, N (%)	Pneumonia, N (%)	Pleural effusion, N (%)	Pneumothorax, N (%)	Chylothorax, N (%)	ARDS, N (%)	Respiratory failure, N (%)	Heart failure, N (%)	Anastomotic fistula, N (%)	Hoarse voice, N (%)	Bleeding, N (%)	Wound infection, N (%)	ICU treatment, N (%)	Die, N (%)
Lee et al,[21] 2019	26	-	-	-	-	-	-	-	-	-	-	-	-	-	-
Zhang et al,[40] 2021	23	-	15 (65.2%)	10 (43.5%)	-	1 (4.3%)	-	-	-	3 (13.0%)	1 (4.3%)	1 (4.3%)	-	-	-
Duan et al,[27] 2021	17	-	6 (35.2%)	-	-	-	2 (11.8%)	2 (11.8%)	4 (23.5%)	2 (11.8%)	5 (29.4%)	-	-	-	-
Xing et al,[28] 2021	11	-	5 (45.5%)	-	-	-	-	-	-	1 (9.1%)	-	-	-	-	-
Huang et al,[29] 2021	21	-	-	-	-	-	-	-	-	-	-	-	-	-	-
Wu et al,[37] 2021	38	10 (26.3%)	9 (23.7%)	-	-	1 (2.9%)	-	-	-	-	-	-	1 (2.9%)	5 (14.3%)	-
He et al,[42] 2021	16	-	-	-	-	-	-	-	-	-	-	-	-	-	-
Gu et al,[23] 2020	15	-	-	-	-	-	-	-	-	-	-	-	-	-	-
Ma et al,[32] 2021	7	-	-	-	-	-	-	-	-	-	-	-	-	-	-
Yan et al,[38] 2021	36	-	-	-	-	-	-	-	-	-	-	-	-	-	-

© 2022 Ge F et al. *JAMA Network Open.*

Sun et al,[45] 2022	26	-	-	-	-	-	-	-	-	-	-	-	-	-	-
Wang et al,[35] 2021	12	-	-	-	-	-	-	-	-	-	-	-	-	-	-
Liu et al,[44] 2022	51	24 (47.1%)	5 9.8%	3 5.9%	2 (3.9%)	4 (7.8%)	-	-	2 (3.9%)	-	13 (25.5 %)	-	-	1 (2.0%)	-
Zhang et al,[41] 2021	40	-	-	-	-	-	-	-	-	-	-	-	-	-	-
Xu et al,[46] 2022	46	-	-	-	-	-	-	-	-	-	-	-	-	-	-
Wang et al,[36] 2021	24	-	-	-	-	-	-	-	-	-	-	-	-	-	-
Liu et al,[31] 2021	28	-	-	-	-	-	-	-	-	1 (3.6%)	-	-	-	-	-
Liu et al,[31] 2021	18	-	-	-	-	-	-	-	-	-	-	-	-	-	-
van den Ende et al,[22] 2019	33	-	10 (30.3%)	-	-	5 (15.2%)	-	-	-	7 (21.2%)	-	-	-	-	-
Yang et al,[47] 2021	20	-	-	-	-	-	-	-	-	2 (10.0%)	1 (5.0%)	1 (5.0%)	1 (5.0%)	-	-
Zhang et al,[25] 2020	18	-	-	-	-	-	-	-	-	-	-	-	-	-	-

© 2022 Ge F et al. *JAMA Network Open.*

Shang et al,[33] 2021	29	-	-	-	-	-	-	-	-	-	-	-	-	-	-
Liu et al,[43] 2021	51	14 (27.5%)	7 (13.7%)	3 (5.9%)	2 (3.9%)	-	-	-	-	3 (5.9%)	5 (9.8%)	-	1 (2.0%)	-	-
Park et al,[24] 2020	16	11 (68.8%)	-	-	-	-	3 (18.8%)	3 (18.8%)	-	3 (18.8%)	3 (18.8%)	-	1 (6.3%)	-	2 (12.5)
Shen et al,[34] 2021	27	-	3 (11.1%)	4 (14.8%)	-	2 (7.4%)	-	-	-	5 (18.5%)	2 (7.4%)	-	-	-	-
Yang et al,[39] 2021	12	2 (16.7%)	-	-	-	-	-	-	-	2 (16.7%)	-	-	-	-	-
Athauda et al,[26] 2021	15	5 (33.3%)	-	-	-	-	-	-	-	1 (6.7%)	-	-	-	-	-
N, number; ARDS, acute respiratory distress syndrome; ICU, intensive care unit															

© 2022 Ge F et al. *JAMA Network Open.*

eFigure 1. The geographical distribution of included studies. Color depth is proportional to the number of included studies, including 22 in China, 2 in South Korea, 1 in Germany, 1 in the Netherlands, and 1 in the USA.

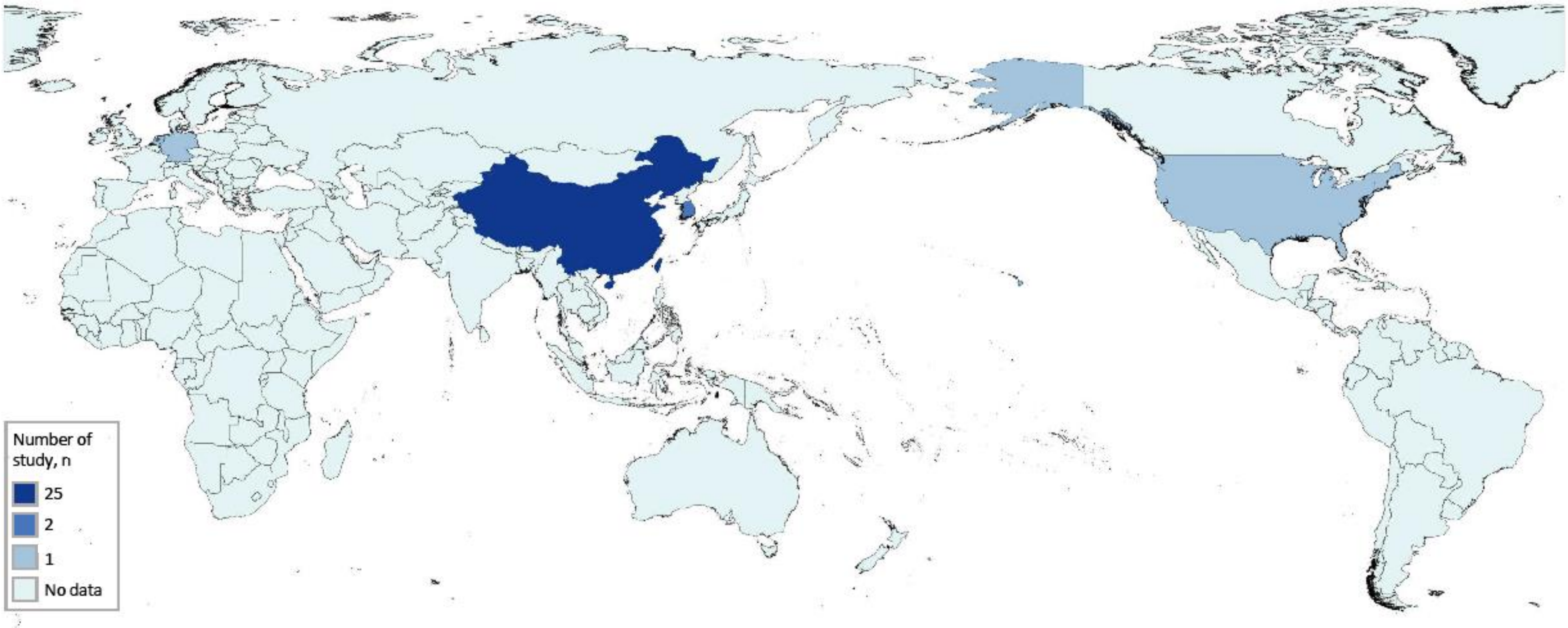

© 2022 Ge F et al. *JAMA Network Open.*

eFigure 2. Methodological quality assessment of included studies. *, For comparative studies only. #, Scores of at least 75% were considered high quality with low risk for bias; scores between 50% and 75% were considered medium risk for bias; scores of less than or equal to 50% were considered high risk for bias. For noncomparative studies, the maximum score was 16, while the maximum score for comparative studies was 24.

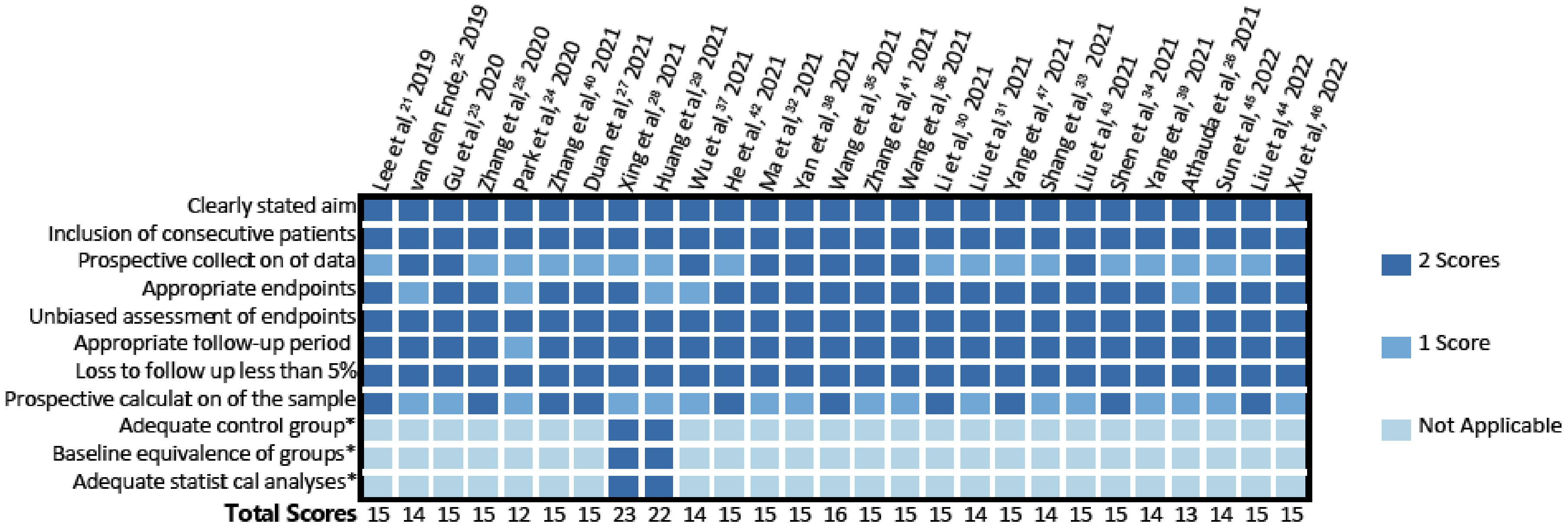

© 2022 Ge F et al. *JAMA Network Open.*

eFigure 3. Forest plot of the primary outcomes for efficacy in neoadjuvant immunotherapy combined with chemotherapy in ESCC stratified by ICI types. (A) the pCR rate, (B) the MPR rate. CI, confidence interval; ESCC, esophageal squamous cell carcinoma; ICI, immune-checkpoint inhibitors; pCR, pathological

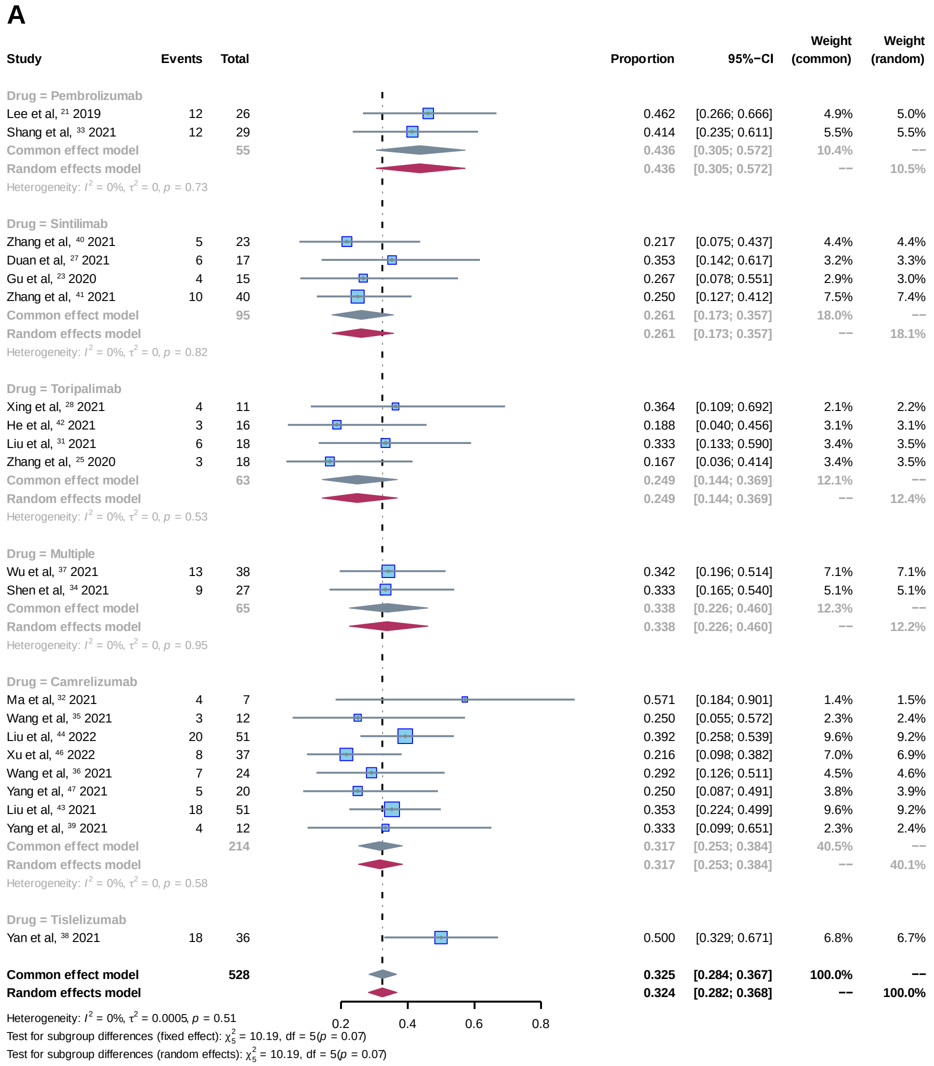

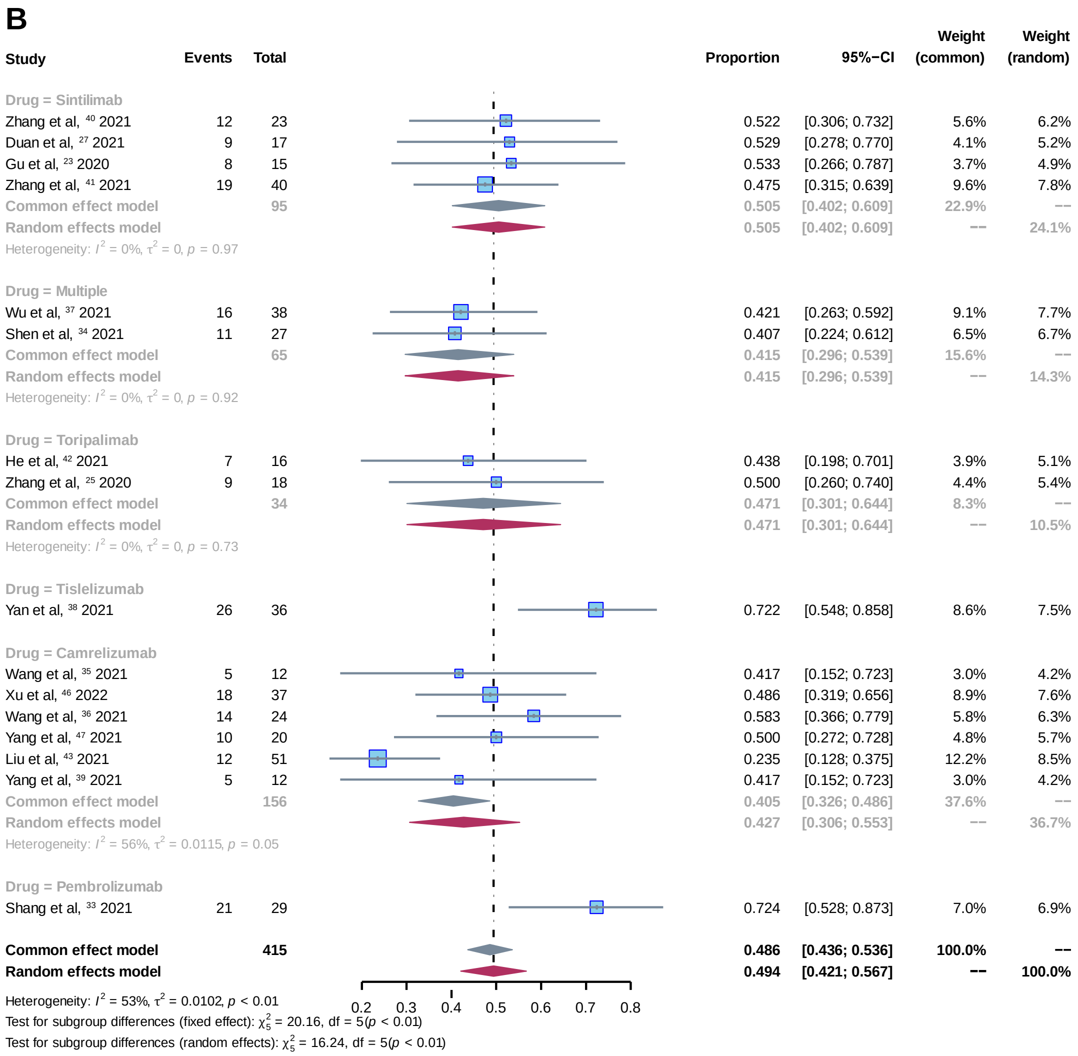

© 2022 Ge F et al. *JAMA Network Open.*

complete response; MPR, major pathological response

eFigure 4. Forest plot of the secondary outcomes for efficacy in neoadjuvant immunotherapy combined with chemotherapy in ESCC stratified by ICI types. (A) CR, (B) PR, (C) SD, (D) ORR, (E) DCR. CI, confidence interval; ESCC, esophageal squamous cell carcinoma; ICI, immune-checkpoint inhibitors; PR, partial response; CR, complete response; SD, stable disease; ORR, objective response rate; DCR, disease control rate

A

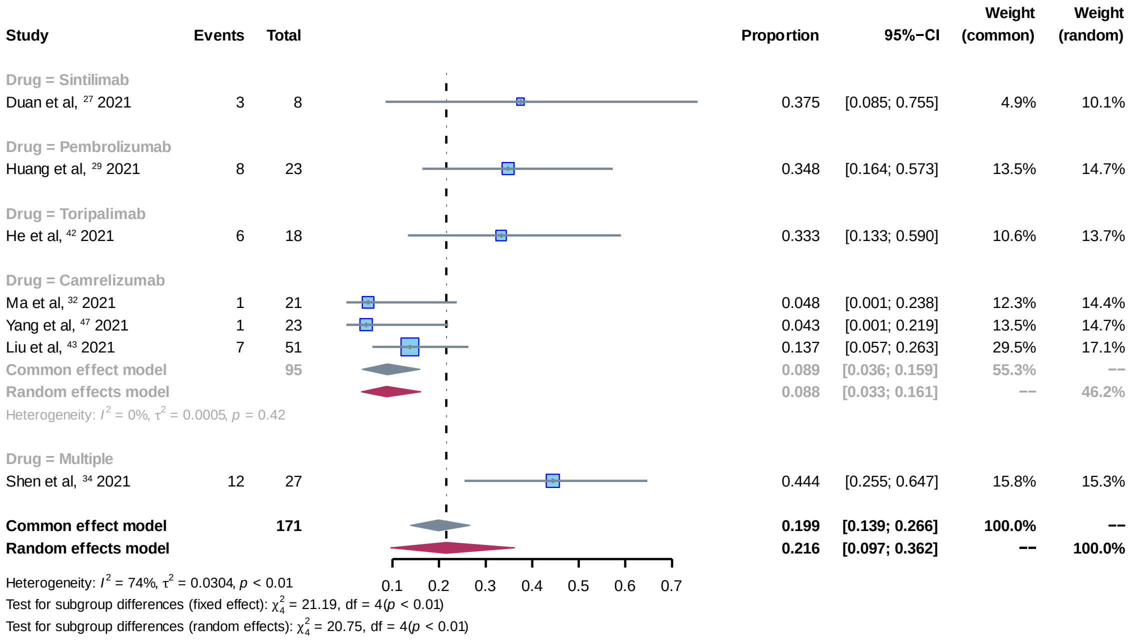

© 2022 Ge F et al. *JAMA Network Open.*

B

Study	Events	Total	Proportion	95%−CI	Weight (common)	Weight (random)
Drug = Sintilimab						
Zhang et al,[40] 2021	20	30	0.667	[0.472; 0.827]	11.7%	10.3%
Duan et al,[27] 2021	2	8	0.250	[0.032; 0.651]	3.3%	5.9%
Common effect model		38	0.583	[0.415; 0.743]	14.9%	--
Random effects model			0.492	[0.121; 0.868]	--	16.1%
Heterogeneity: $I^2 = 76\%$, $\tau^2 = 0.0598$, $p = 0.04$						
Drug = Pembrolizumab						
Huang et al,[29] 2021	12	23	0.522	[0.306; 0.732]	9.0%	9.4%
Drug = Multiple						
Wu et al,[37] 2021	13	19	0.684	[0.434; 0.874]	7.5%	8.8%
Shen et al,[34] 2021	12	27	0.444	[0.255; 0.647]	10.5%	9.9%
Common effect model		46	0.545	[0.396; 0.689]	18.0%	--
Random effects model			0.557	[0.323; 0.779]	--	18.8%
Heterogeneity: $I^2 = 60\%$, $\tau^2 = 0.0161$, $p = 0.12$						
Drug = Toripalimab						
He et al,[42] 2021	5	18	0.278	[0.097; 0.535]	7.1%	8.6%
Zhang et al,[25] 2020	19	24	0.792	[0.578; 0.929]	9.4%	9.6%
Common effect model		42	0.579	[0.423; 0.728]	16.4%	--
Random effects model			0.545	[0.083; 0.964]	--	18.2%
Heterogeneity: $I^2 = 91\%$, $\tau^2 = 0.1209$, $p < 0.01$						
Drug = Camrelizumab						
Ma et al,[32] 2021	7	21	0.333	[0.146; 0.570]	8.2%	9.1%
Yang et al,[47] 2021	18	23	0.783	[0.563; 0.925]	9.0%	9.4%
Liu et al,[43] 2021	27	51	0.529	[0.385; 0.671]	19.7%	11.6%
Yang et al,[39] 2021	7	12	0.583	[0.277; 0.848]	4.8%	7.2%
Common effect model		107	0.555	[0.457; 0.651]	41.7%	--
Random effects model			0.559	[0.371; 0.739]	--	37.5%
Heterogeneity: $I^2 = 68\%$, $\tau^2 = 0.0231$, $p = 0.03$						
**Common effect model**		**256**	**0.558**	**[0.495; 0.621]**	**100.0%**	**--**
**Random effects model**			**0.549**	**[0.438; 0.658]**	**--**	**100.0%**

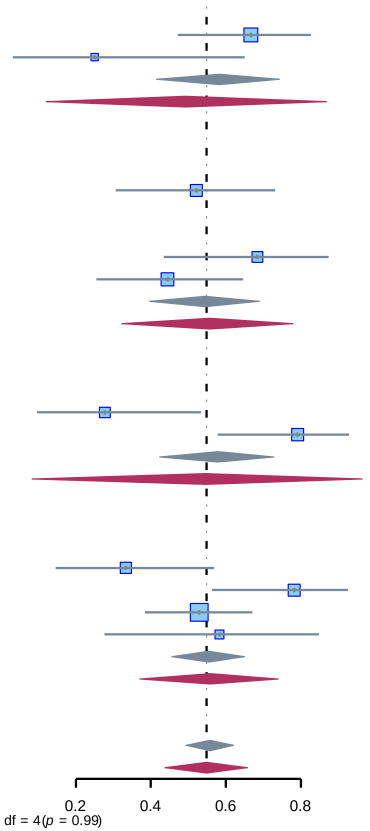

Heterogeneity: $I^2 = 64\%$, $\tau^2 = 0.0202$, $p < 0.01$
Test for subgroup differences (fixed effect): $\chi^2_4 = 0.30$, df = 4 ($p = 0.99$)
Test for subgroup differences (random effects): $\chi^2_4 = 0.14$, df = 4 ($p = 1.00$)

© 2022 Ge F et al. *JAMA Network Open*.

C

Study	Events	Total	Proportion	95%−CI	Weight (common)	Weight (random)
Drug = Sintilimab						
Zhang et al, [40] 2021	9	30	0.300	[0.147; 0.494]	11.7%	9.9%
Duan et al, [27] 2021	2	8	0.250	[0.032; 0.651]	3.3%	6.6%
Common effect model		38	0.284	[0.143; 0.447]	14.9%	−−
Random effects model			0.284	[0.143; 0.447]	−−	16.5%
Heterogeneity: $I^2 = 0\%$, $\tau^2 = 0$, $p = 0.86$						
Drug = Pembrolizumab						
Huang et al, [29] 2021	2	23	0.087	[0.011; 0.280]	9.0%	9.4%
Drug = Multiple						
Wu et al, [37] 2021	6	19	0.316	[0.126; 0.566]	7.5%	8.9%
Shen et al, [34] 2021	3	27	0.111	[0.024; 0.292]	10.5%	9.7%
Common effect model		46	0.186	[0.082; 0.316]	18.0%	−−
Random effects model			0.197	[0.037; 0.428]	−−	18.7%
Heterogeneity: $I^2 = 64\%$, $\tau^2 = 0.0193$, $p = 0.10$						
Drug = Toripalimab						
He et al, [42] 2021	7	18	0.389	[0.173; 0.643]	7.1%	8.8%
Zhang et al, [25] 2020	5	24	0.208	[0.071; 0.422]	9.4%	9.5%
Common effect model		42	0.281	[0.151; 0.431]	16.4%	−−
Random effects model			0.286	[0.127; 0.475]	−−	18.3%
Heterogeneity: $I^2 = 35\%$, $\tau^2 = 0.0065$, $p = 0.21$						
Drug = Camrelizumab						
Ma et al, [32] 2021	13	21	0.619	[0.384; 0.819]	8.2%	9.2%
Yang et al, [47] 2021	0	23	0.000	[0.000; 0.148]	9.0%	9.4%
Liu et al, [43] 2021	17	51	0.333	[0.208; 0.479]	19.7%	10.8%
Yang et al, [39] 2021	3	12	0.250	[0.055; 0.572]	4.8%	7.7%
Common effect model		107	0.267	[0.184; 0.358]	41.7%	−−
Random effects model			0.255	[0.027; 0.585]	−−	37.1%
Heterogeneity: $I^2 = 90\%$, $\tau^2 = 0.0988$, $p < 0.01$						
**Common effect model**		**256**	**0.237**	**[0.184; 0.294]**	**100.0%**	**−−**
**Random effects model**			**0.236**	**[0.131; 0.358]**	**−−**	**100.0%**

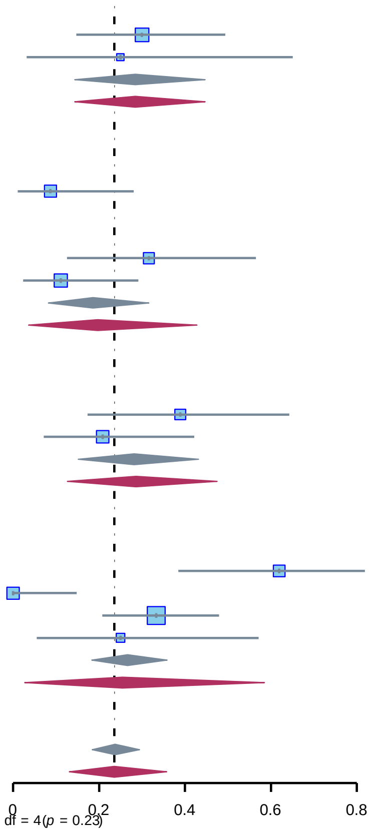

Heterogeneity: $I^2 = 75\%$, $\tau^2 = 0.0334$, $p < 0.01$

Test for subgroup differences (fixed effect): $\chi^2_4 = 5.60$, df = 4($p = 0.23$)

Test for subgroup differences (random effects): $\chi^2_4 = 4.47$, df = 4($p = 0.35$)

© 2022 Ge F et al. *JAMA Network Open.*

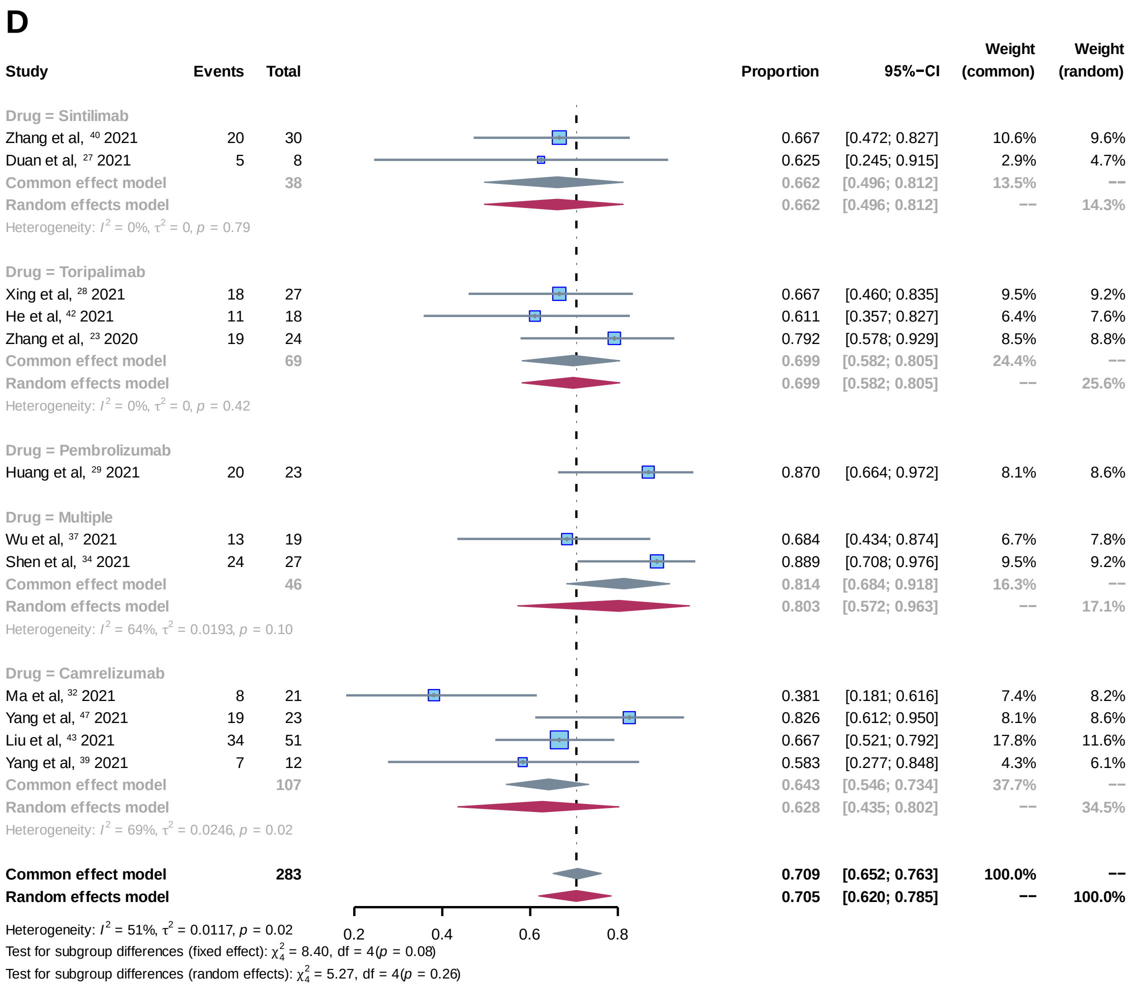

© 2022 Ge F et al. *JAMA Network Open.*

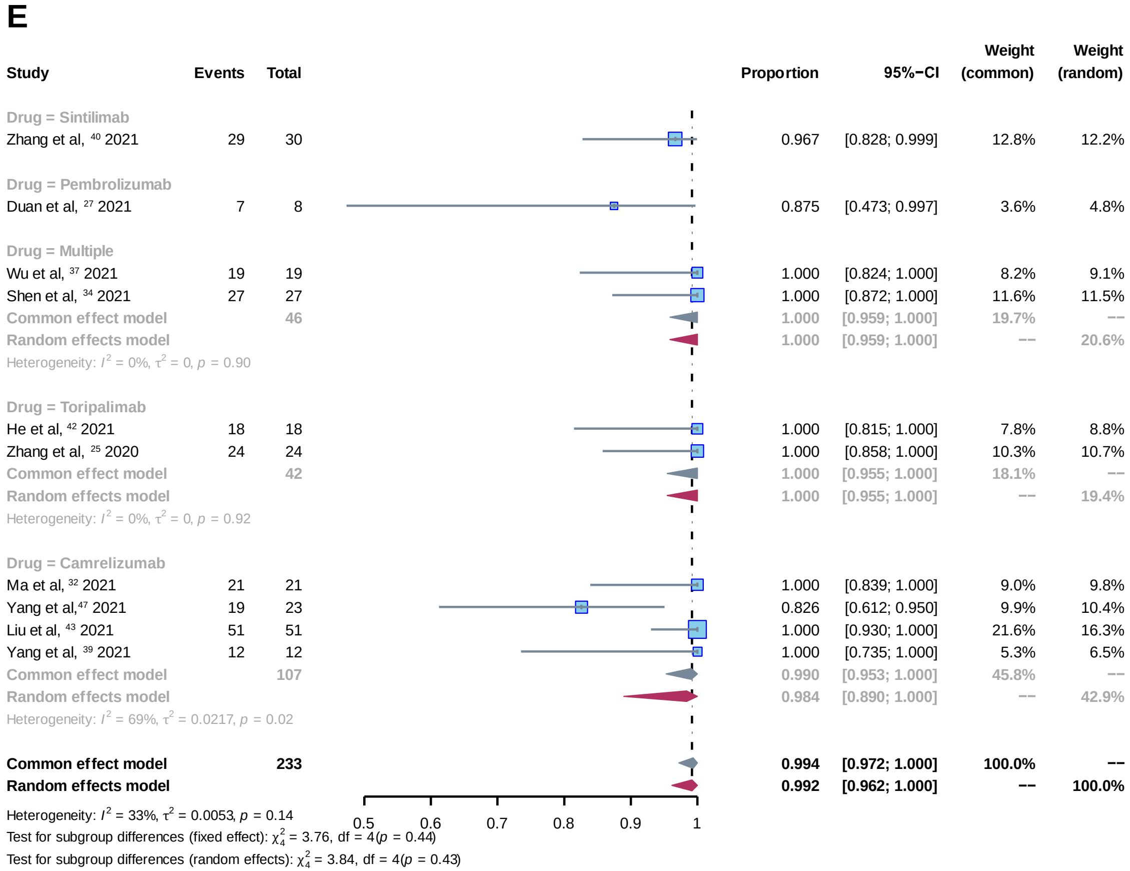

© 2022 Ge F et al. *JAMA Network Open.*

eFigure 5. Forest plot of the outcomes for safety in neoadjuvant immunotherapy combined with chemotherapy in ESCC stratified by ICI types. (A) the incidence of tr-SAE, (B) R0 surgical resection rate. CI, confidence interval; ESCC, esophageal squamous cell carcinoma; ICI, immune-checkpoint inhibitors; tr-SAE, treatment-related severe adverse events

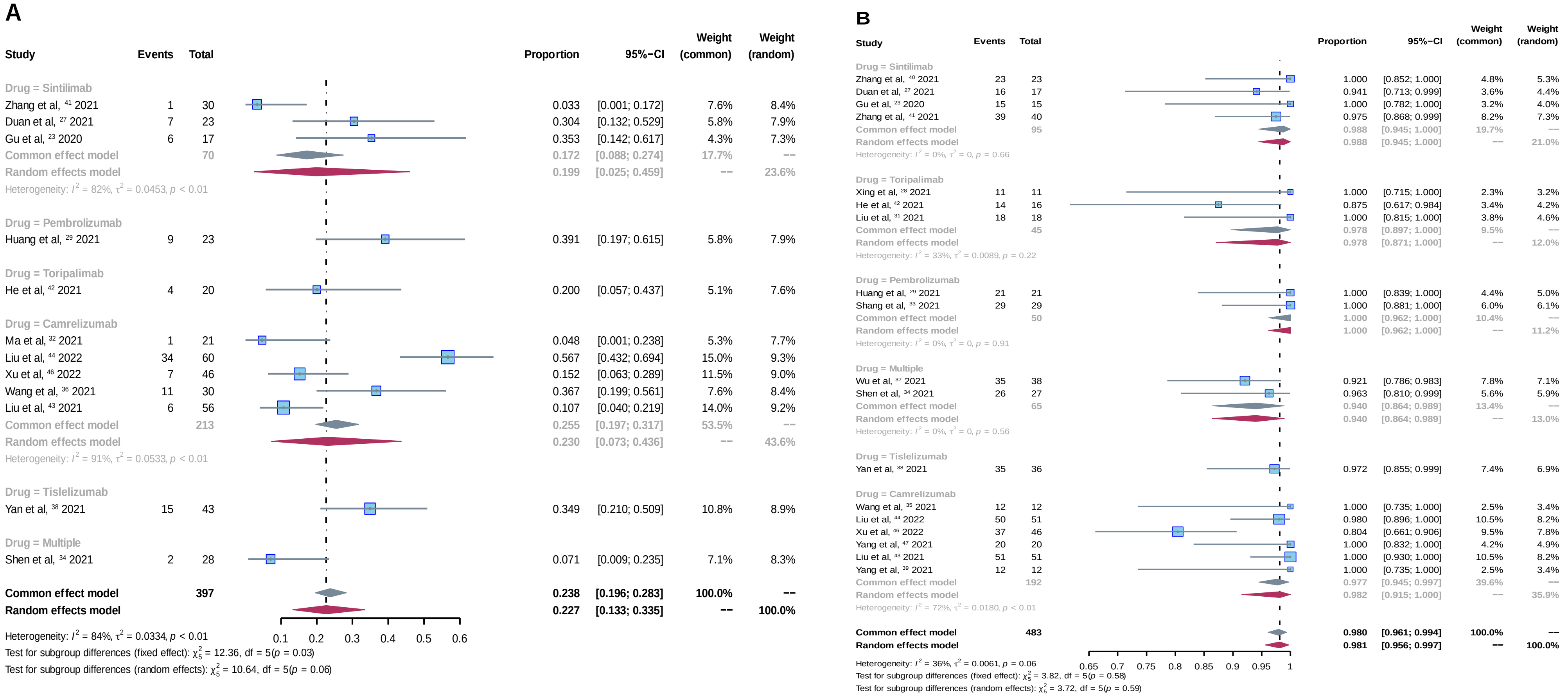

© 2022 Ge F et al. *JAMA Network Open.*

eFigure 6. Sensitivity analyses of the outcomes by repeating the pooled analyses with one study omitted at a time. (A) pCR rate, (B) MPR rate, (C) the incidence of tr-SAE, (D) R0 surgical resection rate. CI, confidence interval; pCR, pathological complete response; MPR, major pathological response; tr-SAE, treatment-related severe adverse events

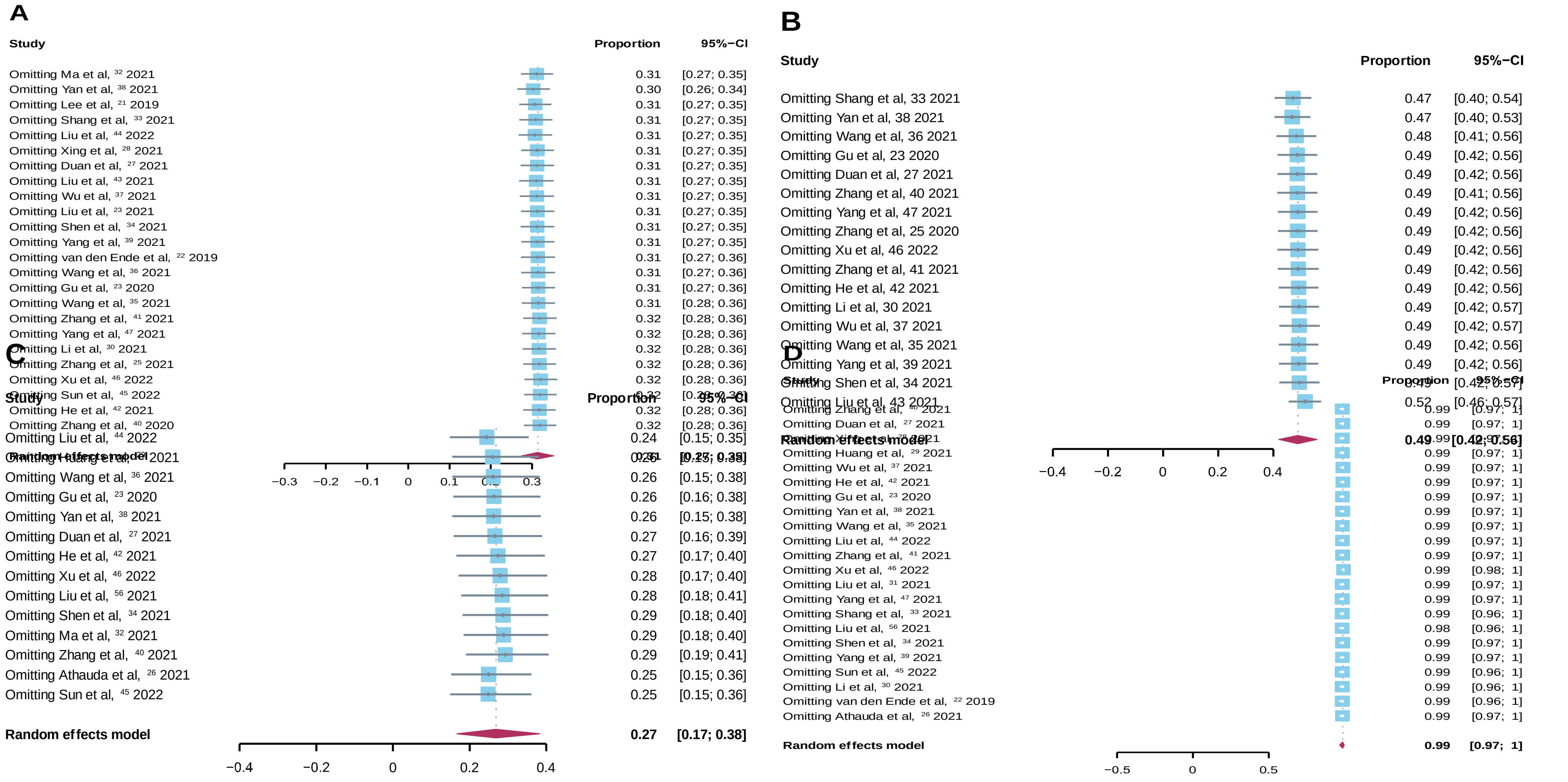

© 2022 Ge F et al. *JAMA Network Open.*

eFigure 7. Sensitivity analyses based on the stage of disease in ESCC. (A) pCR rate, (B) MPR rate, (C) R0 surgical resection rate. (D) ORR, (E) DCR. CI, confidence interval; ESCC, esophageal squamous cell carcinoma; pCR, pathological complete response; MPR, major pathological response; ORR, objective response rate; DCR, disease control rate

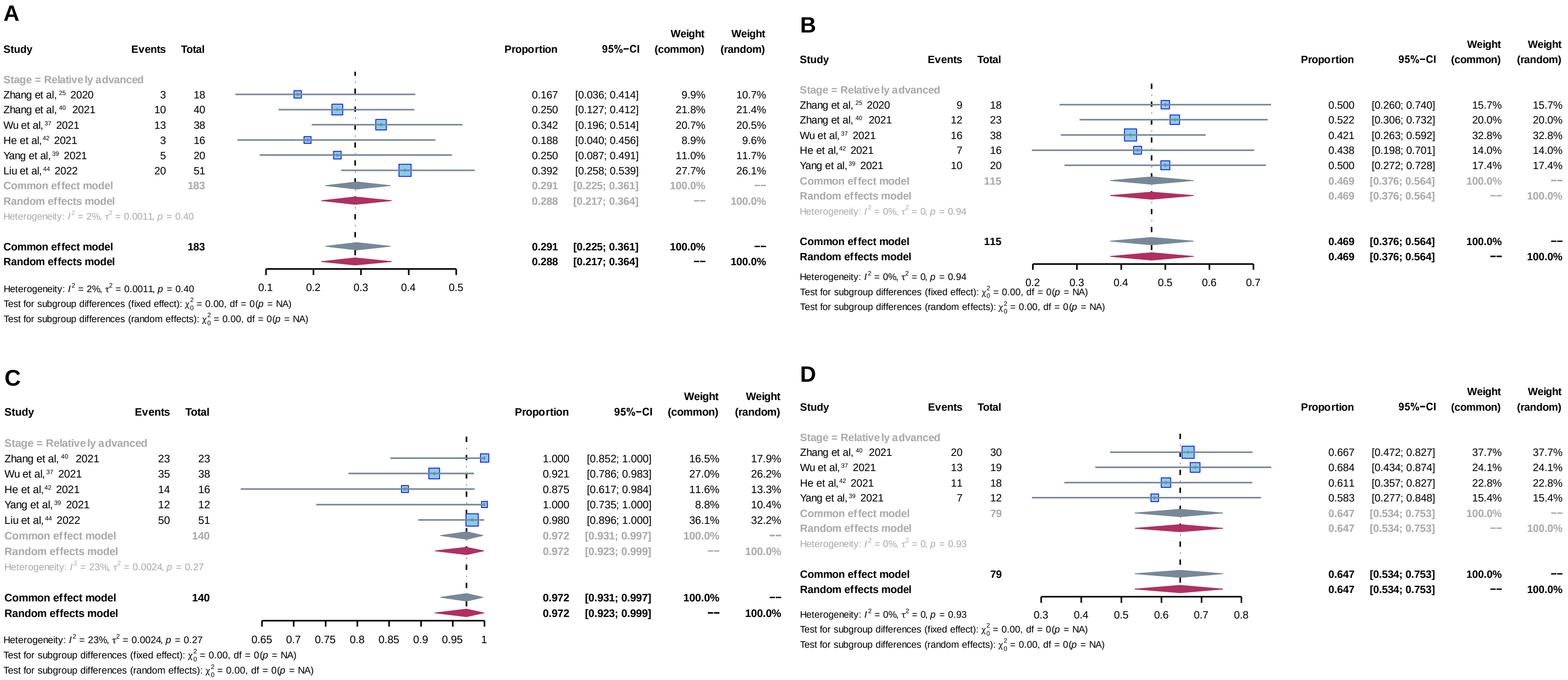

© 2022 Ge F et al. *JAMA Network Open.*

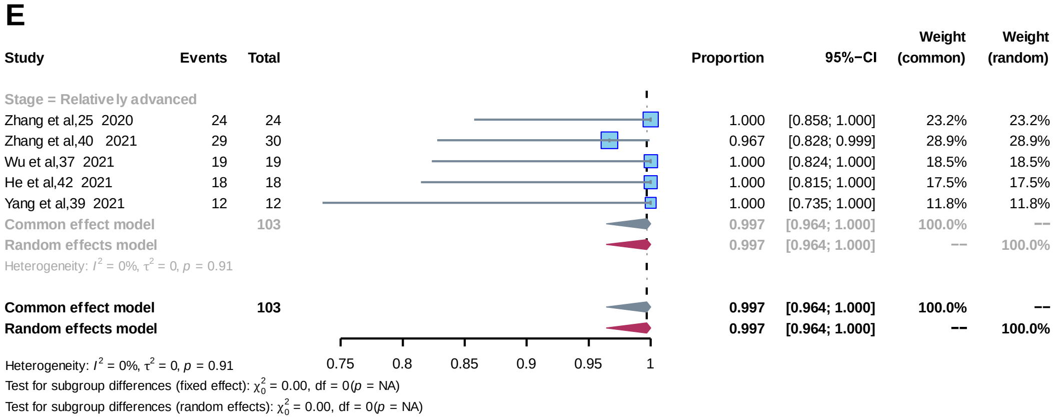

© 2022 Ge F et al. *JAMA Network Open.*

eFigure 8. Egger's tests of the outcomes to detect publication bias. (A) pCR rate, (B) MPR rate, (C) the incidence of tr-SAE, (D) R0 surgical resection rate. CI, confidence interval; pCR, pathological complete response; MPR, major pathological response; tr-SAE, treatment-related severe adverse events

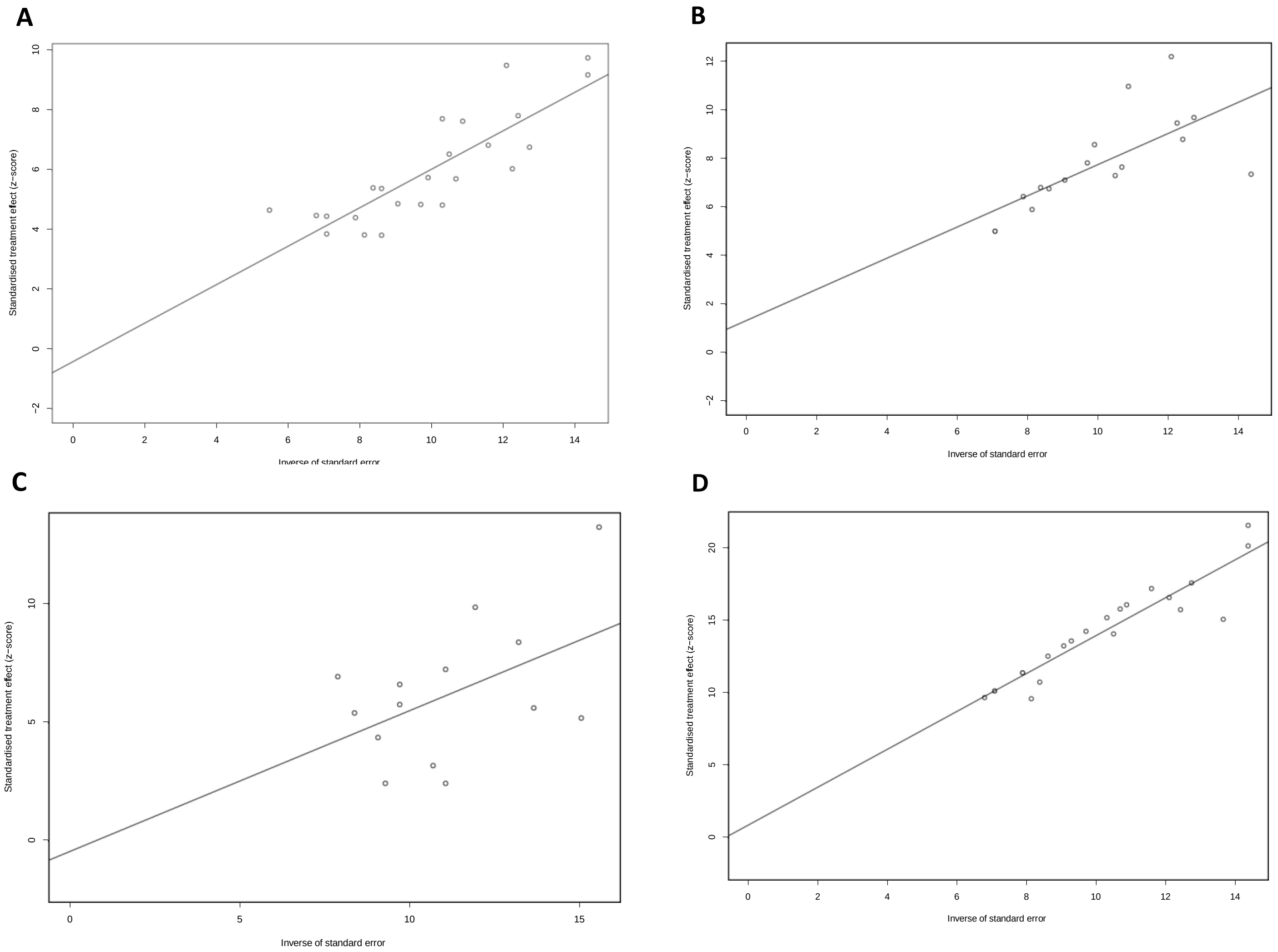

© 2022 Ge F et al. *JAMA Network Open.*

eFigure 9. Forest Plot of Safety Outcomes of Neoadjuvant Immunotherapy Combined With Chemotherapy

A

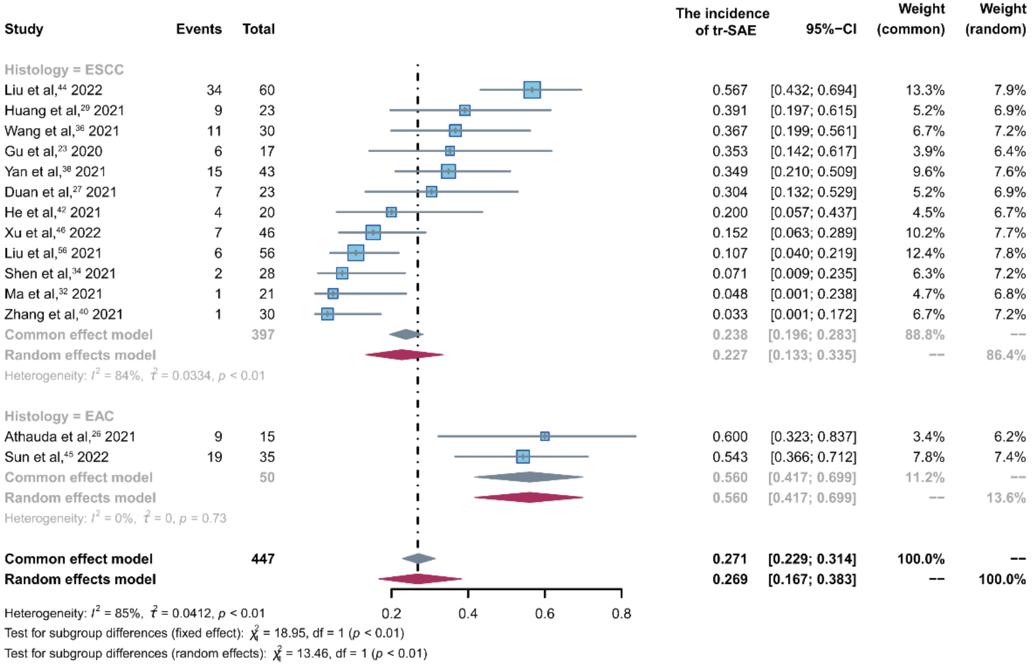

B

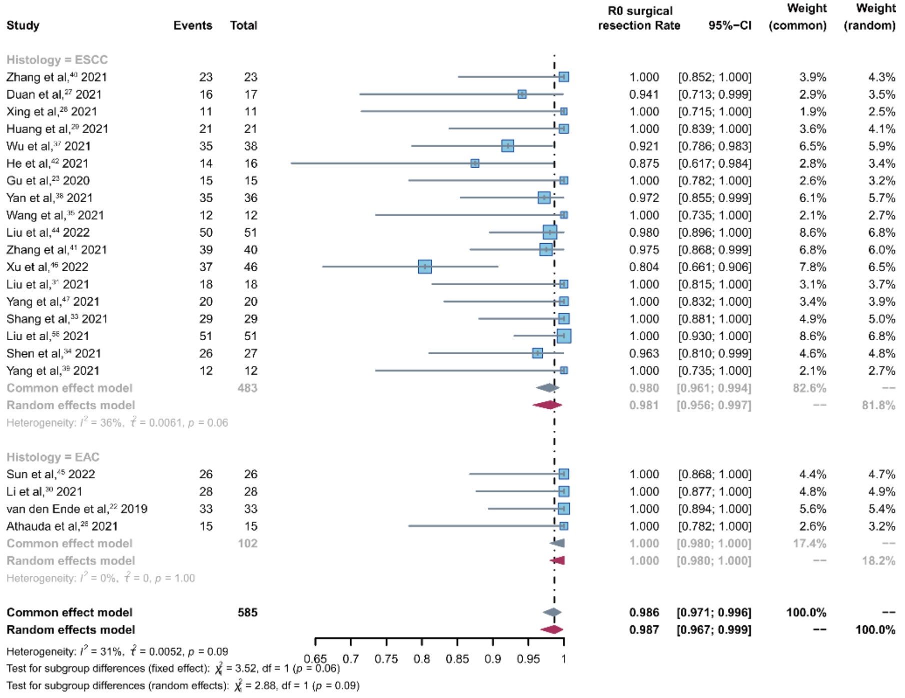

© 2022 Ge F et al. *JAMA Network Open.*
